# Computational Disorder Analysis in Ethylene Response Factors Uncovers Binding Motifs Critical to Their Diverse Functions

**DOI:** 10.3390/ijms21010074

**Published:** 2019-12-20

**Authors:** Xiaolin Sun, Nawar Malhis, Bi Zhao, Bin Xue, Joerg Gsponer, Erik H. A. Rikkerink

**Affiliations:** 1 The New Zealand Institute for Plant & Food Research Ltd., 120 Mt. Albert Rd, Private Bag 92169, 1025 Auckland, New Zealand; xiaolin.sun@plantandfood.co.nz; 2 Michael Smith Laboratories—Centre for High-Throughput Biology, The University of British Columbia, Vancouver, BC V6T 1Z4, Canada; nmalhis@chibi.ubc.ca (N.M.); gsponer@msl.ubc.ca (J.G.); 3 Department of Cell Biology, Microbiology and Molecular Biology, School of Natural Sciences and Mathematics, College of Arts and Sciences, University of South Florida, 4202 East Fowler Avenue, ISA 2015, Tampa, FL 33620-5150, USA; bizhao@mail.usf.edu (B.Z.); binxue@usf.edu (B.X.)

**Keywords:** abiotic and biotic stress, AP2 domain, DNA binding, ERF transcription factors, intrinsically disordered protein, molecular recognition features (MoRFs), transcription activation, transcription repression

## Abstract

APETALA2/ETHYLENE RESPONSE FACTOR transcription factors (AP2/ERFs) play crucial roles in adaptation to stresses such as those caused by pathogens, wounding and cold. Although their name suggests a specific role in ethylene signalling, some ERF members also co-ordinate signals regulated by other key plant stress hormones such as jasmonate, abscisic acid and salicylate. We analysed a set of ERF proteins from three divergent plant species for intrinsically disorder regions containing conserved segments involved in protein–protein interaction known as Molecular Recognition Features (MoRFs). Then we correlated the MoRFs identified with a number of known functional features where these could be identified. Our analyses suggest that MoRFs, with plasticity in their disordered surroundings, are highly functional and may have been shuffled between related protein families driven by selection. A particularly important role may be played by the alpha helical component of the structured DNA binding domain to permit specificity. We also present examples of computationally identified MoRFs that have no known function and provide a valuable conceptual framework to link both disordered and ordered structural features within this family to diverse function.

## 1. Introduction

Plants are constantly challenged to survive under environmental stresses. Abiotic stresses such as drought, salinity, and extreme temperatures influence plant growth and development by altering phytohormonal balance and redox processes, potentially leading to damage [1]. On the other hand, biotic stress stimulates the synthesis of phytohormones like salicylic acid (SA), jasmonic acid (JA), and ethylene (ET), which regulate specific immune responses [2]. Plants respond and adapt to these environmental stresses by triggering molecular signal transduction cascades to rapidly fine-tune their metabolic status in order to maintain homeostasis. Plant hormones function in these cascades as central integrators, reprogramming complex stress-adaptive signalling cascades [3]. Among the range of plant response events, these phytohormones are responsible for transcriptional changes that enable physiological adaptation and trigger plant disease resistance. Transcription factors (TFs) are essential components of the integration of hormone signalling pathways in transcriptional relays.

APETALA2/ETHYLENE RESPONSE FACTOR (AP2/ERF) transcription factors (ERFs), one of the largest families of the plant-specific TFs, play a pivotal role in adaptation to biotic and abiotic stresses such as those caused by pathogens, wounding, and salinity by acting as key integrators of the SA, JA, and ET stress signalling pathways [4]. Plants contain a large array of ERFs, consisting of 122 proteins in Arabidopsis (At) and 139 in rice (Os) [5]. Each ERF protein contains a highly conserved DNA-binding domain (DBD or AP2 domain), which consists of about 57–59 amino acid residues. Many ERFs have been shown to bind specifically to the GCC box (AGCCGCC), a core sequence essential for ethylene-responsive transcription of genes, and are associated with biotic stress responses. While other ERFs bind to the C-repeat/dehydration-responsive-element (CRT/DRE; A/GCCGAC) cis-acting sequence in the promoters of target genes and are usually associated with responses to abiotic stress [6]. TFs recognize specific DNA sequences through their DBD, but typically recruit and assemble key transcription machinery partners through their transcription regulatory domains (TRDs). These two components integrate the protein-DNA and protein–protein recognition events that are central to function. Intrinsically disordered proteins (IDPs) or regions (IDR) are over-represented in proteins located in the nucleus and prevalent in cell signalling and transcriptional regulation [7,8]. IDRs are often characterized by the unique combination of high specificity and low affinity in their interactions with functional partners, which is important for transient protein–protein and protein–nucleic acid interactions occurring during signal transduction, recognition, and regulation events. Disorder also leads to binding promiscuity that enables one IDR to interact with multiple structurally diverse partners and binding plasticity through Molecular Recognition Features (MoRFs, an interaction prone segment within a long-disordered region). These MoRFs initiate recognition upon binding to various specific partners. Together these factors facilitate specific recognition by IDPs of their biological targets, allowing the formation of a flexible interaction network and conferring functional advantages on IDPs operating in stress response, signalling and regulation [9,10,11].

Bioinformatic studies revealed that 94% to 82% of the TFs possess IDRs as well as numerous MoRFs mainly found within their TRDs, relative to 54% to 18% of the proteins in two control data sets [8]. Experimental studies of TF families such as NAC and bZIP have demonstrated that IDRs dominate in their regulatory domains, providing the necessary structural flexibility for the TFs to interact with other TFs and/or signalling proteins in interaction networks and sense a wide variety of intracellular and extracellular stimuli [12,13]. MoRFs contained in these IDRs are conserved in each sub-group of the TFs families as similar binding motifs, enabling the functionalisation of TF subgroups by interacting with specific proteins of transcriptional complexes using either one or more of these specific motifs. Such mechanisms can exert fine-tuned transcriptional control over signalling pathways and sometimes involve specific binding-induced folding of the MoRFs [13]. Recent studies across a number of plant species have identified interesting correlations (positive or negative) between cellular complexity and an overall increase in disorder in some transcription factor families (e.g., MYB and bZIP) but not in other families such as ERFs [14,15]. ERFs are involved in a wide variety of biological functions in response to both biotic and abiotic stresses, and are required for cross-talk between signalling pathways crucial for adapting to environmental challenges. We present an intrinsic disorder analysis of ERFs from three divergent plant species, Arabidopsis, rice and moss. A number of computational approaches determined the degree of disorder or structure along the length of the ERF protein sequences and detected conserved patterns of disorder, MoRFs and ordered regions that could be correlated with ERF proteins in general or specific ERF subfamilies in particular. Functional specificities for some of the subgroups are correlated with their respective conserved MoRFs in TRDs. Local structural flexibility in DBDs of ERFs which, this analysis indicates, may be another key factor contributing to the functional specificity of individual members, could also help explain the plethora of stress phenotypes influenced by this large plant-specific disordered family. We illustrate the biological significance of these computational analyses with examples from phenotypes as diverse as cold stress, cytokinin control, hypoxia, and pathogen response, where they correlate with protein motifs identified as critical components of the control exerted by these versatile proteins.

## 2. Results and Discussion

### 2.1. Phylogenetic Analysis and Classification of ERF Families

**Figure 1:**
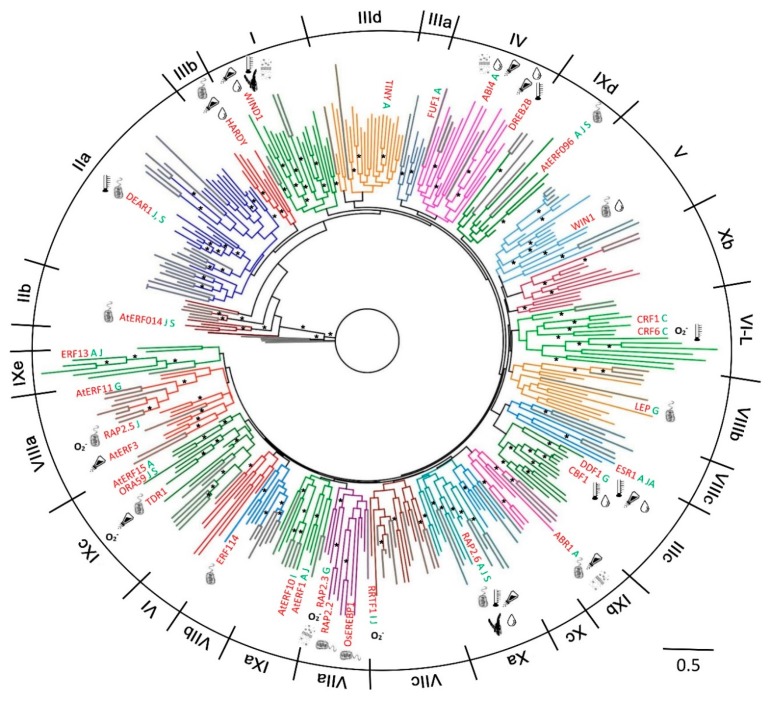
Phylogenetic tree of 375 ETHYLENE RESPONSE FACTOR transcription factors (ERFs) from Arabidopsis, rice and moss. The groups/subgroups are indicated in Roman numbers. Some key proteins with functional data are given for each of the subgroups where we could identify these and the types of stress they are associated with is also given to help orient the reader with respect to groups of related genes with functional data. Groups and sub-groups were initially identified from a tree including all members from all species. Given that the gene family is so diverse the support for the group and subgroup branches at the base of this tree was sometimes insufficient (less than 50%) to be confident that the grouping reflects the most likely phylogeny. Similar trees presented in the previous published analyses have often not shown any values for the support of groupings, branches with greater than 70% support are indicated by an asterisk. Angiosperm branches are coloured by clade with moss branches indicated in grey. Keys: hormones (single letter text after gene name)—A, Abscisic acid; C, Cytokinin; G, gibberellic acid; I, IAA/auxin; J, Jasmonic acid; S, salicylic acid. Stress symbols associated with individual identified genes (inside the circle) or un-identified genes in the subgroup (outside of the circle)– Biotic 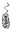; Cold/Heat 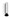; Osmotic 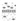; Oxidative 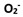; Salt 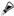; Water/Drought 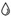; Wounding 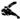.

**Figure 2:**
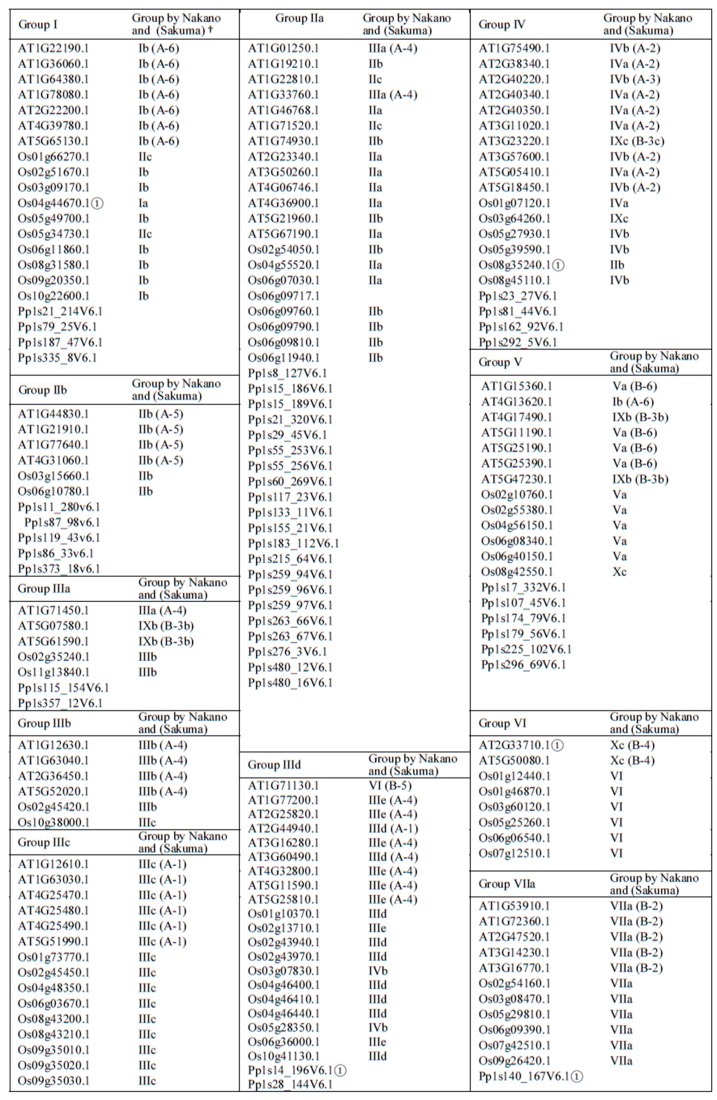

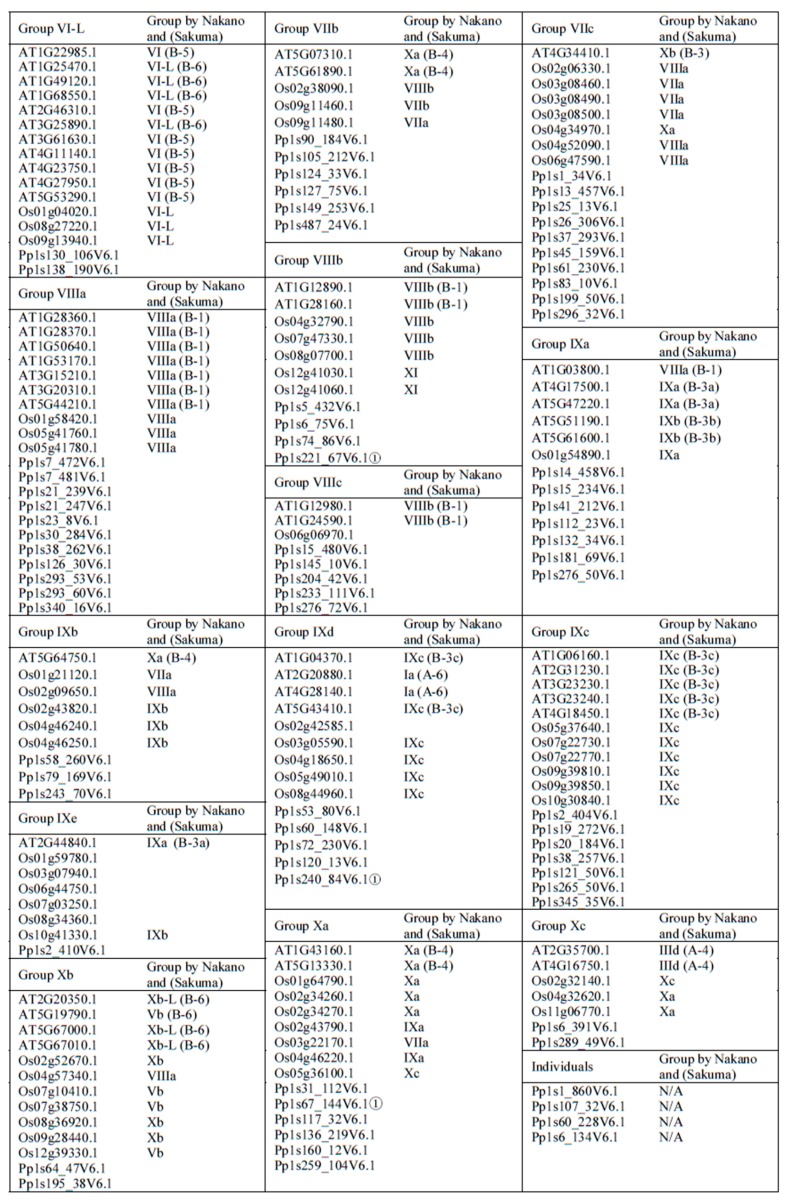
Groups of ERFs. More distantly related genes that have uncertain group membership status are labelled ①. On the phylogenetic trees they occupy a position that is basal to the rest of the members of the group. They have also been tested for membership of other groups but appear not to fit with any other group. ^†^ Grouping of this ERF by Nakano et al. (2006) where it exists while its grouping by Sakuma et al. (2002) where it exists is given in brackets.

To generate a family tree and classify proteins into ERF families, a total of 375 ERF sequences from three different plant species were included in a phylogenetic analysis based on multiple alignment of the AP2/ERF domains and re-alignment of the regions outside of the AP2/ERF domains (Figure 1 and Figure 2). Based on this family tree, these ERFs can be classified into 10 groups and 25 subgroups, not including four individual Moss ERFs which may represent other new groups. The resultant classification is mostly consistent with earlier reports of Arabidopsis and rice ERFs [5] with some subgroups refined. As shown in Figure 2, many of the ERFs classified previously still cluster in the same group, e.g., most of IIb, IIIb, IIIc, VIIa, VIIIa and IXc group members remain in the same groups. Some minor re-classifications are reported in Figure 2 (e.g., some members previously in IIb have moved to IIa, previous subgroups IIId and IIIe are combined into subgroup IIId, and IVa and IVb members are combined into subgroup IV). These refinements are largely based on the conserved motifs in regions outside of the ERF domain aligned under the subgroup classification.

Phylogenetic classification of the big ERF family is complicated by the highly variable sequences in the regions outside of the AP2/ERF domains while the high degree of similarity in the rather short conserved AP2/ERF domains reduces the statistical confidence in critical basal branch points. We suggest that this combination reduces the power of normal phylogenetic approaches to illustrate the relationship between groups or subgroups of genes. Hence the fact that some of the individual ERFs sequences have been reclassified into different subgroups compared to previous classifications [5,6] is not surprising. In order to provide more confidence in these groupings we therefore tested the support for each subgroup in sub-trees with the same outgroup (Pp1s107_32V6.1) to verify the final groupings. Isolated proteins that were positioned basal to the other members of the group identified by this approach were re-tested for membership with other families. As a result, a few ERFs were moved between groups or subgroups after this analysis, while no new grouping could be identified for others and these were retained as distant members of the group (identified in Figure 2). Four representative examples of these subgroupings complete with their branch support values are shown in Supplemental Figure S1. There are limitations to the degree to which we can predict functional and protein interaction characteristics for ERFs by depending on sequence alignment alone, therefore we subjected the 375 ERFs sequences to disorder and MoRF predictions to investigate the protein interaction characteristics in their 10 groups (25 subgroups). The members of each group/subgroup together with their alternative names and functions where available are listed in Supplementary Table S1.

### 2.2. Multiple Analyses Show that the TRDs Domain of ERF Proteins are Intrinsically Disordered

**Figure 3:**
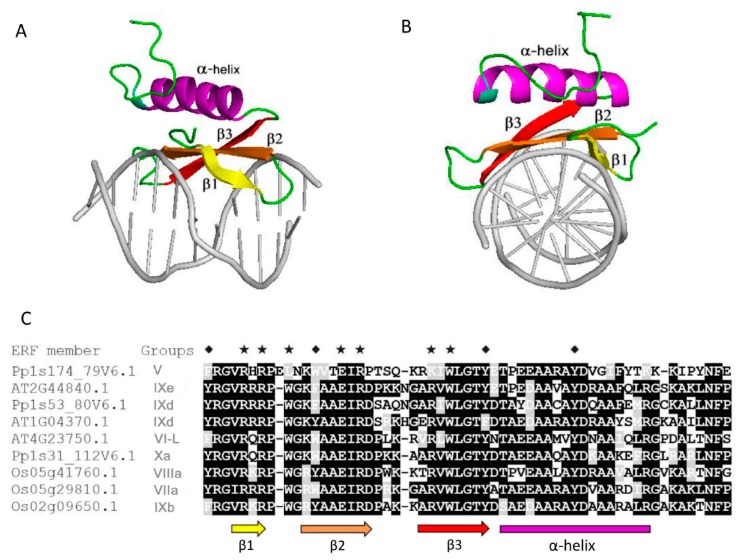
Structure of the DNA binding AP2 domain of AtERF1 complexed with DNA and sequence alignments. (**A**) Horizontal perspective. (**B**) Perpendicular perspective. (**C**) Sequence alignment of AP2 domain sequences randomly selected from a set of groups and different plant species (Arabidopsis, rice and moss). Black shadings indicate identical residues, grey shadings indicate conservatively substituted residues. The coloured arrows and bar represent β-strands and α-helix regions, respectively. The asterisks represent residues directly contacting with DNA. The diamonds represent highly conserved tyrosine or conservatively substituted tyrosine residues discussed in the text. The structural figures were prepared using PyMOL software with PDB code 1GCC.

Previously, it was demonstrated that most TFs are intrinsically disordered in their TRDs [8,13]. We carried out multiple bioinformatics analyses using our ERF protein dataset. This revealed similar results to previous findings on other TFs in that all of the TRDs of the ERFs are intrinsically disordered while the DBDs largely consist of folded structures (Figure 3A,B). This had already been confirmed experimentally by NMR spectrometry in one example [16]. Within the DBDs of AtERF1 the first three β-strands are highly ordered while the following α-helical regions are relatively flexible.

**Figure 4:**
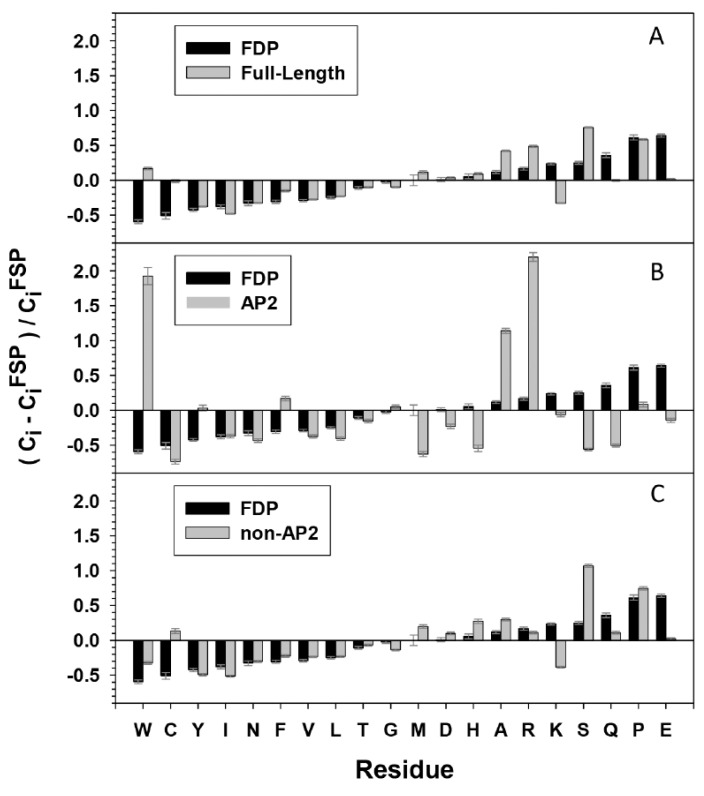
Relative compositional profile of amino acids of the 375 ERF sequences compared to Fully Structured Proteins (FSP). For (**A**) full-length ERF proteins, (**B**) AP2 domains only, and (**C**) AP2 domain-deleted ERF proteins. FDP shows the amino acids composition of Fully Disordered Proteins (FDP) compared to FSP. Probability distributions were estimated by computing means and confidence intervals of the relative frequencies of residues observed over a set of pseudo-replicate datasets obtained by bootstrap sampling of whole proteins from the original samples, the bootstrapping was repeated 10,000 times. The amino acid residues are placed along a continuous scale from the greatest contribution to ordered structure (W, left) to the greatest contribution to disorder (E, right).

The propensity for disorder is encoded in the composition of amino acid sequences. Low overall hydrophobicity and high net charge are widely used as one of the criteria for IDPs predictions [17,18]. Amino acid compositional profiles of ERF families from the three species analysed are similar to those typical of intrinsically disordered proteins. This is the case for full length sequences, but is even more pronounced when the mainly structured AP2 domain (DBD) is omitted (Figure 4A,C). The ERF sequences generally show a lack of order-promoting residues and an enrichment in disorder-promoting residues. The exception to the trend for over-representation of disorder promoting residues is an overall lower content of lysine residues in the disordered TRD regions, a unique compositional feature to be investigated. In contrast, the AP2 domains within the ERFs display significant differences to the compositional profile of IDPs and several residues that vary in amino acids composition compared to fully structured proteins (Figure 4B). The AP2 domains utilize an unusually high portion of W, A and R compared to both structured and disordered proteins. We deduce from sequences of the AP2 domains (Figure 3C) that these three residues, especially R, are frequently used for direct contact of DNA bases or for structural framing throughout the ordered AP2 domains.

Charge and hydropathy plot (CH-plot) of a protein can be used as a linear protein disorder classifier that differentiates proteins with substantial amounts of disorder from proteins with globular conformations [18,19]. Another binary disorder classifier, cumulative distribution function (CDF) analysis, differentiates all proteins containing any IDRs from those that contain mainly folded domains [20]. Simultaneous CDF-CH plotting provides an even more accurate prediction for a wider range of proteins [21]. The quadrants of CDF-CH phase space correspond to the following expectations: Q1 (upper-right), proteins predicted to be disordered by CH-plots, but ordered by CDFs; Q2 (lower-right), ordered proteins in terms of both methods; Q3 (lower-left), proteins predicted to be disordered by CDFs, but compact by CH-plots; Q4 (upper-left), proteins predicted to be disordered by both methods.

**Figure 5:**
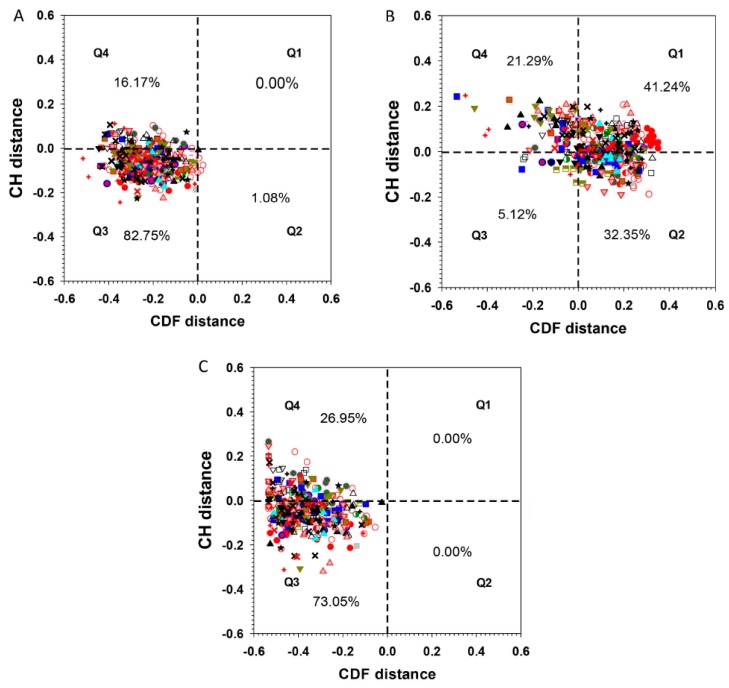
Charge-hydropathy and cumulative distribution function (CH-CDF) plot. For (**A**) full-length ERF proteins, (**B**) ERF-AP2 domains only, and (**C**) AP2-deleted ERF proteins. The percentages indicate total number of ERFs in each of the quadrant. The symbols representing the members of each group/subgroup: I 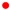, IIa 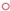, IIb 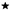, IIIa 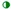, IIIb 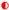, IIIc 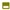, IIId 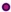, IV 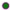, V 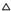, VI-L 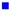, VI 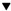, VIIa 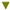, VIIb 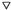, VIIc 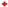, VIIIa 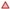, VIIIb 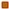, VIIIc 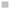, IXa 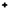, IXb 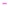, IXc 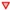, IXd 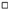, IXe 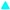, Xa 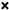, Xb 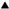, Xc 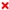.

Full-length ERF proteins all cluster in the quadrants Q4 and Q3 of the CH-CDF phase space (Figure 5A). The AP2 domains as a set move to the right of this plot to occupy space between Q1 and Q2 (Figure 5B), while the AP2 domain-deleted regions as a set move further to the left within Q4 and Q3 (Figure 5C). This demonstrates that the TRDs of the ERFs proteins (located outside of the AP2 domain) are intrinsically disordered while most of the AP2 domains (DBD) are predicted to be ordered in terms of the CDF criterion. On the other hand, more than half of full-length ERFs and the AP2 domain-deleted regions have CH distances located in Q3 while the rest of the AP2 domain regions have CH distances falling in quadrant Q4. CH-plots by themselves appear not to be a suitable disorder predictor in the case of ERF proteins, indicating that charge and hydropathy are not the dominant factors driving their structural disorder. This is also reflected in the fact that the folded AP2 domains (DBD) contain a high portion of R, E and D polar residues interspersed within a frame formed by conserved Y, W and F that facilitate DNA binding.

**Figure 6:**
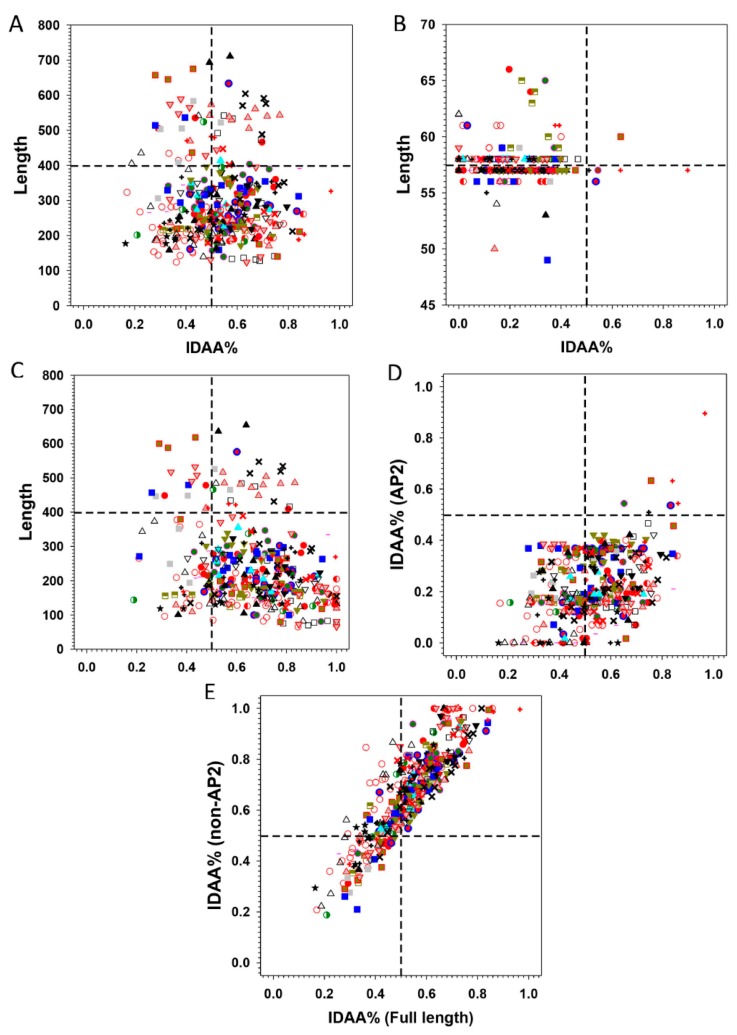
Comparison of different sequence features with the fraction of intrinsically disordered amino acids (IDAA%). (**A**) Length distribution of full-length ERFs, (**B**) Length distribution of AP2 domains, (**C**) Length distribution of AP2-deleted ERFs, (**D**) Comparison of the IDAA% between full-length and AP2 domains, (**E**) Comparison of the IDAA% between full-length proteins and AP2-deleted ERFs. The symbols representing the members of each group/subgroup: I 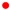, IIa 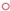, IIb 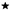, IIIa 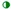, IIIb 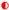, IIIc 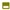, IIId 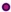, IV 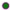, V 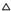, VI-L 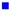, VI 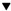, VIIa 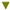, VIIb 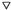, VIIc 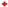, VIIIa 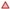, VIIIb 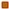, VIIIc 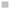, IXa 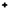, IXb 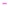, IXc 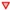, IXd 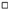, IXe 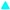, Xa 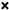, Xb 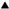, Xc 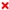.

Statistics on the fraction of intrinsically disorder (promoting) amino acids (IDAA%) for both full-length, AP2 domains alone, and the AP2 domains-deleted ERFs are given in Figure 6. These figures reveal that the non-AP2 regions of ERFs host the majority of disorder promoting residues, with IDAA% spanning from 30% to 80% in the case of full length sequences and 40% to 100% in the case of AP2 domains-deleted sequences only (Figure 6A,C), in comparison with 0% to 40% in the case of AP2 domains alone (Figure 6B). In addition, the differential correlation of IDAA% between the AP2 domains alone (Figure 6D), AP2 domains-deleted (Figure 6E) and full-length sequences of ERFs also show that the non-AP2 regions are the predominant determinant of intrinsic disorder propensity for ERFs.

**Figure 7:**
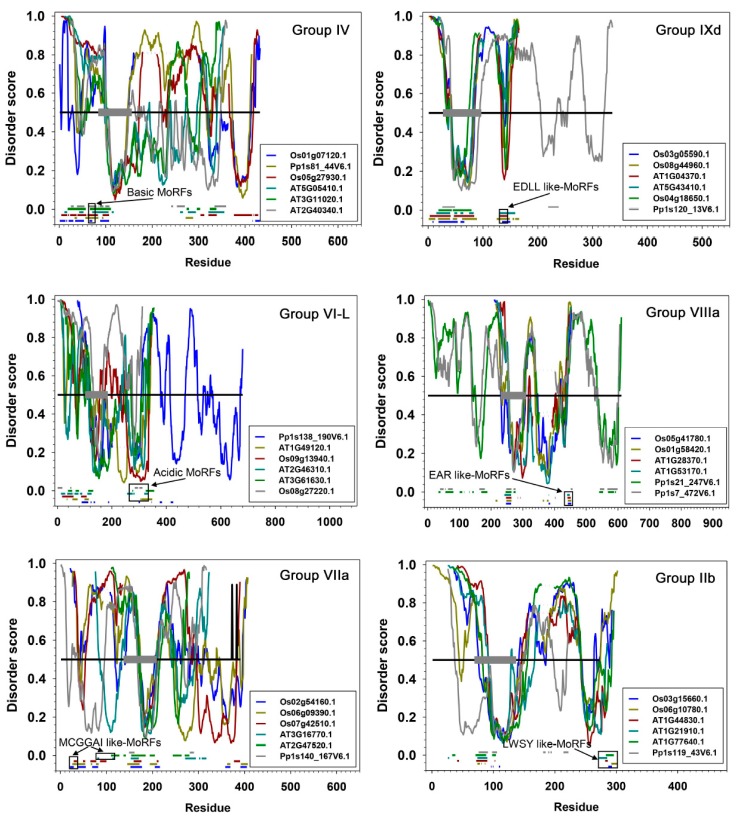
Disorder prediction of ERFs. Six groups with six representative members selected in each group are presented while all groups are presented in Supplemental Figure S2. Disorder score was predicted using PONDR-FIT. The disorder curves were aligned using IDAlign. The grey thick bars indicate the span of the AP2 (DBD) domains including the three highly structured beta sheets and a less ordered alpha helix at the C-terminal end of this domain. The black horizontal line represents the disorder threshold score (≥0.5). The short horizontal colour bars at the bottom of each panel are Molecular Recognition Features (MoRFs) predicted using MoRFchibi. In each group/subgroup presented, arrows point at a specific type of MoRF shown in the black boxes.

Distribution of the intrinsic disorder propensities along the length of sequences for each subgroup are shown in PONDR-FIT plots (Figure 7 and Supplemental Figure S2). Six members in each subgroup are presented to illustrate the overall distribution pattern of disordered residues and MoRFs identified by using MoRFchibi. The DNA binding AP2 domains marked by grey thick bars are universally predicted to consist of mainly ordered segments (Figure 7), i.e., structurally folded zone. The regions outside of the AP2 domains, harbouring TRDs, are generally located in the disordered zone (disorder score >0.5 threshold). Frequent downward spikes representing local short segments with order propensity within the long-disordered region and generally coincide with MoRFs (marked by various short coloured bars) that typically act as binding sites in the interactions with partners during transcriptional regulation. These results reinforce the findings that ERFs have folded and highly conserved AP2 domains, flanked on both sides by intrinsically disordered regions containing TRDs. Solution structures solved by 2D NMR spectrometry [16] reveal that these AP2 domains fold in three β-strands followed by an α-helix (Figure 3A,B). Experimental evidence from plant NAC and bZIP TFs show a similar general arrangement with the ordered DBDs flanked by the disordered TRDs [12,22].

**Figure 8:**
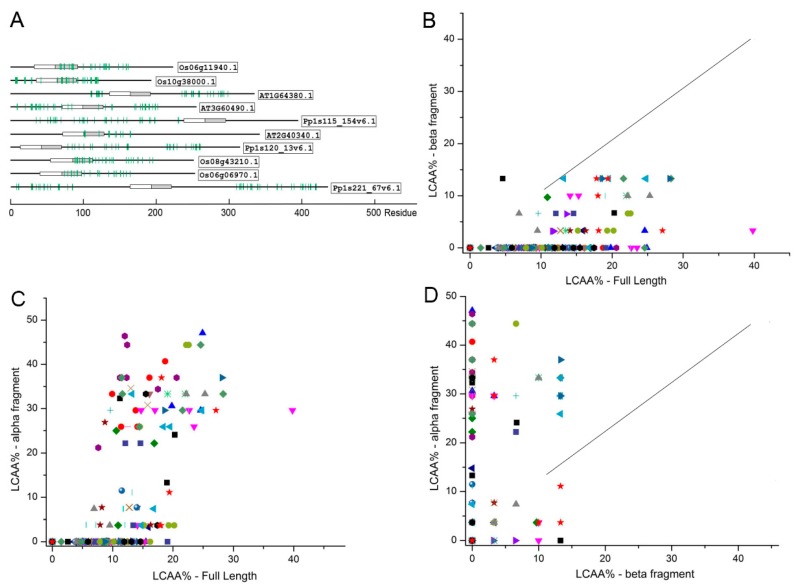
Low complexity analyses of 233 ERF sequences. Low complexity analysis using all members of the small groups and 10 members of each of the large groups. (**A**) Low complexity of one ERF sequence selected randomly from 10 groups/subgroups. Green checks mark the segments of low complexity. (**B**) Percentage of low complexity amino acids (low complexity amino acids (LCAA)%) of full length plotted against LCAA% of beta strands region. (**C**) LCAA% of full length plotted against LCAA% of the APETALA2 (AP2)/ERF domain alpha helix region. (**D**) LCAA% of the AP2/ERF domain beta strands region plotted against LCAA% of the AP2/ERF domain alpha helix region. The symbols representing the members of each group/subgroup: I 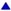, IIa 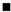, IIb 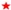, IIIa 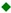, IIIb 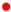, IIIc 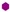, IIId 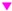, IV 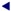, V 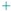, VI-L 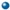, VI 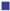, VIIa 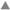, VIIb 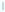, VIIc 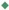, VIIIa 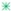, VIIIb 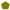, VIIIc 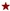, IXa 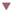, IXb 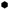, IXc 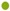, IXd 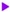, IXe 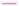, Xa 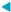, Xb 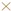, Xc 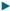.

IDRs and low complexity sequences have similar compositional bias—more disorder-promoting residues and less order-promoting residues [17,23]. Simultaneous use of sequence complexity analysis and intrinsic disorder predictions provides a better view of disorder of sequence segments. Using an iterative algorithm for low complexity analysis (CAST) [24], a total of 233 ERFs sequences from all of the small groups/subgroups and 10 of each large groups/subgroups were selected to determine the distribution of regions of low complexity. The segments with low complexity amino acids (LCAA) in all ERFs selected are predominantly distributed within the long disordered non-AP2 regions (Figure 8A). Commonly, these segments with low complexity contain homo-polymeric stretches of disorder-promoting amino acids. Interestingly, plotting LCAA%-full length against both LCAA%-beta sheet fragments and LCAA%-alpha helix fragments derived from the AP2/ERF domain clearly shows a significant degree of low complexity sequences exist in most α-helical regions within these domains, despite the majority of the low complexity sequences occurring in the non-AP2 regions (Figure 8B–D). This low complexity difference between the beta sheets and alpha helices is also consistent with the sequence alignment (Figure 3C) that show the beta sheet regions generally contain more highly conserved residues and less variable residues than the alpha helical regions. This low complexity distribution indicates that the DNA binding AP2 domains of ERFs can be further divided into an ordered beta sheets region and one much less ordered alpha helical region. This indicates potential functional differences in the DNA binding of these regions that may link to different binding specificities or strengths. Moreover, the structural flexibility of the amino acid across the different parts of AP2 domains may contribute to divergence of DNA binding capacities, promoting flexibility in stress regulation.

### 2.3. Phosphorylation/Dephosphorylation is Involved in ERF Protein Interactions

IDPs manipulate molecular recognition and signalling cascades by utilizing posttranslational modifications (PTMs) such as phosphorylation. For example, phosphorylation has been postulated to (1) fine-tune the electrostatic interactions of disordered regions in protein complexes [25]; to drive folding of an IDP as a regulatory switch [26]; (2) decide between two tiers of the plant immune system as it copes with attacks by type III effectors through a serine/threonine phosphoswitch of the disordered RIN4 protein [27]; and (3) activate the GA signalling pathway by dephosphorylation of serine/threonine residues and phosphorylation of tyrosine residues of the disordered DELLA proteins prior to triggering GA-induced DELLA protein degradation [28]. Compared to structured regions, IDRs possess a much higher probability of phosphorylation, partly attributable to the greater steric access for kinases/phosphatases in open disordered regions and the high portion of disorder-promoting serine residues in these regions [29]. Some of these principles apply equally to other types of PTMs and so disorder facilitates a plethora of alternative protein states to regulate complicated interaction networks and explains why these proteins can occupy pivotal hub positions.

**Figure 9:**
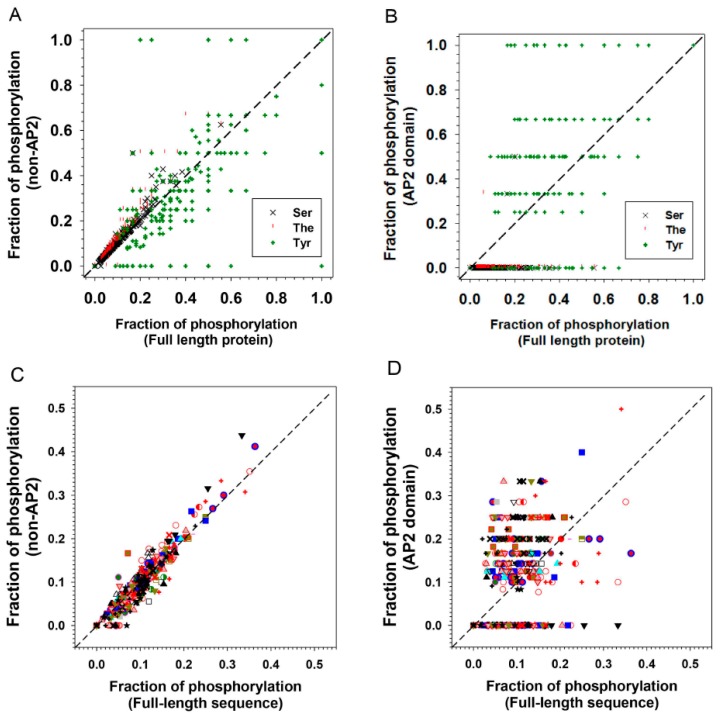
Distribution of phosphorylation sites of 375 ERF proteins. The phosphorylation sites were predicted using MuSite. (**A**) Comparison of fractions of predicted phosphorylation site for each ERF in all groups/subgroups between full-length sequence and AP2-deleted ERF and (**B**) between full-length sequence and AP2 domains. (**C**) Comparison of fractions of predicted phosphorylation site for all three types (Ser, The, Tyr) in all ERF sequences between full-length sequence and AP2-deleted ERFs and (**D**) between full-length sequence and AP2 domains. The symbols representing the members of each group/subgroup: I 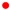, IIa 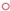, IIb 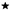, IIIa 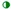, IIIb 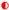, IIIc 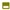, IIId 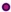, IV 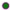, V 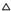, VI-L 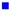, VI 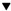, VIIa 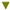, VIIb 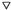, VIIc 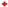, VIIIa 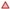, VIIIb 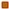, VIIIc 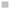, IXa 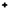, IXb 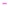, IXc 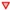, IXd 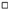, IXe 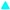, Xa 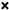, Xb 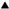, Xc 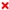.

Phosphorylation is already known to play a role in the function of ERF proteins. A DREB2A ortholog in the grain crop *Pennisetum glaucum* differentially interacts with DNA when an uncharacterised threonine residue is modified and allows it to play a role in the abiotic response in this stress tolerant plant [30]. Two Ser–Pro sites in the C-terminus of AtERF104 are specifically phosphorylated by the MAP kinase MPK6 and increase its stability [31]. Tyrosine sites in the N- and C-terminus of AtERF13 (now deduced to be disordered) were identified to be phosphorylated by cysteine-rich receptor like kinases AtCRK2 and AtCRK3 [32], and AtERF13 is linked with the ABA response [33]. Disorder-based phosphorylation analyses using MuSite for the ERF proteins show that the disordered non-AP2 domain regions display a higher fraction of predicted phosphorylation sites than the full-length sequences and this is true for serine and threonine residues (Figure 9A and Table 1). This result is similar to most other plant IDP families studied [34]. Conversely the ordered AP2 domains of ERFs showed an unexpectedly high proportion of tyrosine residues prone to phosphorylation when compared to the non-AP2 domain regions (Figure 9B). These ordered regions showed a lower proportion of serine/threonine residues prone to phosphorylation. This indicates it is likely that tyrosine phosphorylation plays a special role in AP2 domains in altering DNA binding specificities. As a result of this unusual high portion of tyrosine phosphorylation, the sum of all three types of potential phosphorylation sites (S, T and Y) offsets the S/T phosphorylation preferences in the non-AP2 regions (Figure 9C) and uplift the total potential phosphorylation sites in AP2 domain regions versus full length sequences (Figure 9D). Four notable tyrosine residues in the AP2 domain regions are marked with diamonds in Figure 3C, three occur within the beta sheets and one in the middle of the alpha helix. Interestingly the tyrosines occurring in the alpha helical region are highly conserved whereas those in the more structured core of the beta sheets can be conservatively substituted by other aromatic residues. In the beta sheet cases we postulate that aromatic side chain of these residues play a role in DNA binding through aromatic ring interaction with spatially nearby sidechains from other residues, which has been exemplified in the structure of AtERF1 [16]. These interactions either do not feature phosphorylation or phosphorylation of tyrosine residues introduces further binding or functional diversity between the different families or subfamilies. In contrast, phosphorylation of the highly conserved tyrosines in the less ordered alpha helical region could either encourage or discourage a more extensive DNA interaction with the alpha helix that could be used to fine tune regulation by increasing or decreasing the strength of binding or altering its equilibrium state. The alpha helical fragment in the AP2 domain structure of AtERF1 (Figure 3) is capped on the beta sheet through a hydrogen bond between the hydroxyl group of tyrosine 186 in the α helix and arginine 147 in the β1 strand, implying the potential impact of phosphorylation of tyrosine 186 on the helical structure and interaction between the helix and the beta strand and (we suggest) the resultant DNA binding specificities. Our analyses indicate that future experimental investigation should focuses on these tyrosine residues and how they utilize phosphorylation to affect DNA binding.

### 2.4. Analyses of MoRFs in ERF Groups and Their Functional Implications

In addition to the DNA-binding AP2 domains, ERFs contain functionally important regions outside of AP2 domains, regulating transcriptional activation and protein–protein interactions, defined as the TRD. Generally, many short conserved amino acids motifs are often identified in this region for plant transcription factors such as MYB, WRKY, NAC and GRAS [11,35,36]. ERFs are no exception in this regard. In particular the *Arabidopsis thaliana* ERF family has been found to possess 53 conserved motifs distributed in the regions outside of the AP2 domains across all of the ERF groups/subgroups as determined by sequence alignments [5]. However, the functions of most of these conserved motifs are unknown and there are relatively few clues about which of these motifs are important and what kind of functions they may be important for. Given that ERFs are IDPs with long IDRs in the TRDs regions, it is plausible that analyses for MoRFs can provide a unique way to specifically identify the conserved motifs in these regions that act as key interacting sites in transcriptional regulation. As revealed in our previous studies, MoRFs are often conserved within a subgroup, indicating that members in the same subgroup sharing these MoRFs have similar functions [34]. We carried out MoRFs analyses for all 375 selected ERFs and identified conserved MoRFs (potential interacting sites for partners) in each of the subgroups.

**Figure 10:**
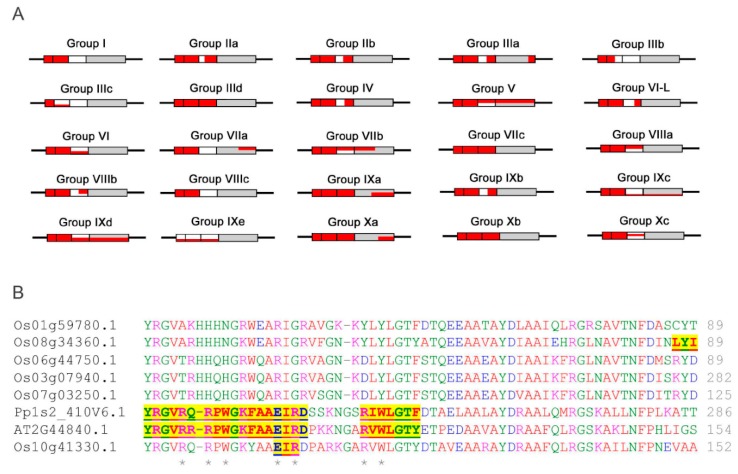
(**A**) Schematic representation of conserved MoRFs in the DNA binding AP2 domains of the ERF groups/subgroups. The β1, β2 and β3 regions are indicated as a set of three boxes followed by the α-helix regions (grey box). The red colour represents conserved MoRFs distributed in each of the groups/subgroups and their location with respect to the β sheets and α helices. Partial red colouring versus white (in β sheets), or grey (in α-helices), reflects incomplete prevalence of MoRFs among members of the groups/subgroups. (**B**) More detailed sequence alignment of the AP2 domains of subgroup IXe. The yellow highlighted regions are MoRFs identified for the three members with DNA contacting residues marked by asterisks. Additional alignments of AP2/ERF regions in other subgroups are shown in Supplemental Figure S3.

The DNA binding AP2 domains in most subgroups of the ERFs family have been found to contain MoRFs (Figure 10A). The MoRFs identified in the AP2 domains are found within nearly all of the β1 and β2 strands, meaning that the β1 and β2 strands are the major binding region in response to interaction between the ERFs and DNA moiety. This is supported by the fact that more than 70% of the direct DNA contact residues (R, E and W) are in the β1 and β2 strand regions and the rest of the direct DNA contact residues are in the β3 strand. This highlights the reliability of the new algorithm MoRFchibi employed in this analysis. Despite being in the DNA binding AP2 domain, the latter parts of the AP2 domains which harbour the α-helix generally lack MoRFs. On the other hand, these α-helical regions have been shown to be more structurally flexible with significant low complexity and more variable sequences. These results are consistent with the observation from the NMR solution structure (Figure 3) that the α-helical region of AtERF1 impacts on ERF DNA binding indirectly rather than by direct binding. We note some exceptions that in three subgroups (V, IXc and IXd) MoRFs can be identified across the whole AP2 domain, indicating that the α-helix may also be involved in direct DNA binding in these individual cases. In contrast, the five rice ERFs in subgroup IXe have no MoRFs identified within their AP2 domains and possess unusual sequence arrangements in which a stretches of multiple histidine residues are inserted in between the β1 and β2 strands while a highly conserved glutamate residue (typically involved in direct contact with DNA) is missing (Figure 10B). To contribute to the divergent DNA-Binding specificities of ERFs, it would be worth investigating experimentally how this clade of ERFs bind DNA. Alternatively, if they do not bind DNA, whether they are involved in other ways such as competing for limited protein complex partners with ERFs that do in fact bind DNA. This same group contains the Arabidopsis AtERF13 (AT2G44840) protein referred to above that is subject to phosphorylation and involved in ABA response.

It has been shown that the DNA binding basic regions in the bZIP transcription factor family, in the absence of DNA, populate an ensemble of highly dynamic transient structures, either completely structured, containing a certain amount of helical structure, or being completely disordered [13]. Experimental evidence revealed that the basic regions of bZIP uniformly form α-helical conformations once in complex with DNA [37]. Our MoRFs and low complexity analyses suggest that the latter part of AP2 domains of ERFs could also be flexible or even disordered in the unbound state, although this region has been shown to be folded as an α-helix when bound with DNA in the case of AtERF1 [16].

**Figure 11:**
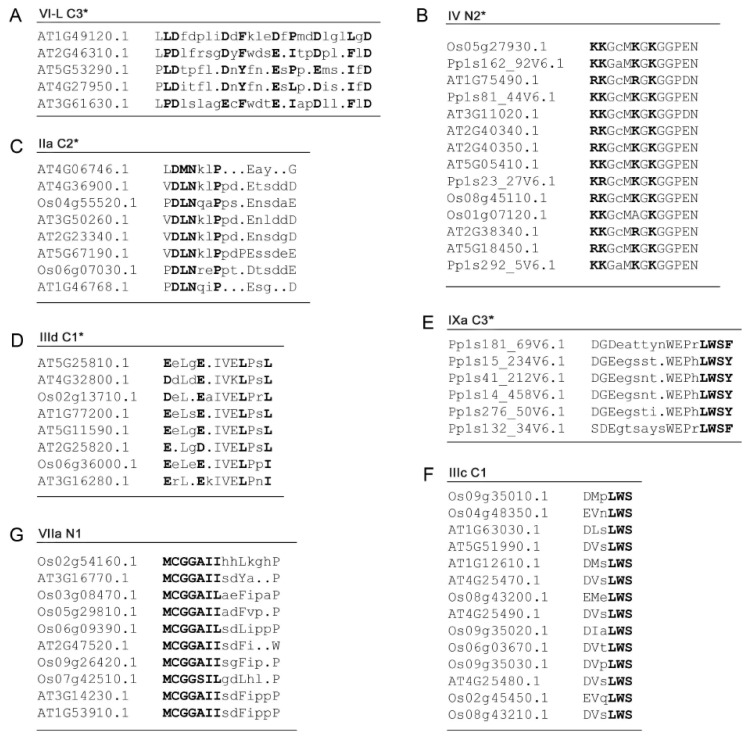
Examples of conserved MoRFs predicted outside of AP2 domains. (**A**) Acidic MoRFs, (**B**) Basic MoRFs, (**C**) ERF-associated amphiphilic repression (EAR) motif-like MoRFs, (**D**) EDLL motif-like MoRFs, (**E**) and (**F**) LWSY motif-like MoRFs, (**G**) MCGGAI(I/L) motif-like MoRFs. The consensus residues of each patterns are in bold. The name of each MoRFs starts with group/subgroup number followed by N (prior to AP2 domain) or C (after AP2 domain), asterisks indicate that this MoRF exclusively belongs to this group/subgroup.

All of the MoRFs identified outside of the AP2 domains are shown in Supplemental Table S2. Many of these are exclusive to specific subgroups in the phylogenetic tree, and likely contribute to similarity in protein interactions within the same subgroup. Although the functions of most of these subgroup-conserved MoRFs are yet to be investigated, the functional roles of some examples that correlate with sites important for transcriptional regulation are discussed below. Many of the conserved MoRFs identified in this study have unique features in their amino acid compositions (Supplemental Table S2). The most common pattern is that either hydrophobic or aromatic residues repeat to form the framework of the MoRFs with acidic residues flanking the repeated hydrophobic/aromatic residues (Figure 11A, also see IIId C2, V C2, VI-L C1*, VI-L C4, VIIa N3, VIIIa N2*, VIIIa C3*, IXb N1*, IXb C2*, and Xb C2* in Supplemental Table S2). Being associated with transcriptional activation [38], such patterns of acidic MoRFs have also been found in other IDPs from the GRAS family. In GRAS proteins the repeated hydrophobic/aromatic residues interspersed between acidic residues have been experimentally demonstrated to directly bind to partners (as in the DELLA subfamily; [34,39,40]), or are responsible for strong transcriptional activation as in the LISCL subfamily; [41]. We postulate that the repeated hydrophobic/aromatic residues together with the acidic residues play similar functional roles in ERFs. Some positively charged residue repeats are identified in the conserved MoRFs of ERFs as well, mostly located in the N-termini of the ERFs (Figure 11B, also see IIIc N1*, VIIIa N4* and IXc C1 in Supplemental Table S2), which are also likely to be involved in specific functions for these groups. Further experimental investigation of these unique patterns of MoRFs is now warranted as this analysis has provided a short-cut to identify likely sites for elucidating the structure-function paradigms of these various ERF families.

Among the subgroup-conserved MoRFs, some overlap directly with motifs that have been experimentally studied. For example three C-terminal ERF-associated amphiphilic repression (EAR) motif-like MoRFs (Figure 11C, also see VIIb C3* and VIIIa C1 in Supplemental Table S2) have been identified and the EAR motifs have been shown to repress GCC box-mediated transcription via transient assays [42,43]. Three EDLL motif-like MoRFs (Figure 11D, also see IXd C1 and IXc C2 in Supplemental Table S2) have been identified where acidic glutamic and aspartic residues are interspersed with hydrophobic leucine residues, constituting acidic transcriptional activation domains (TAD) often found at the C-terminus of transcription factors [44,45]. The EDLL motif has the ability to activate transcription and can confer activation domain function on heterologous DNA-binding proteins [46]. Six LWSY motif-like MoRFs (Figure 11E,F, also see IIa C5, IIb C2*, IIIb C1, and VIIa C2* in Supplemental Table S2) are found to be conserved at the end of the C-terminus of many ERF proteins, they are mostly DREB genes encoding transcription activators that recognise the C-repeat elements of cold-responsive genes (CORs), function in regulation of drought, cold, and salinity responsive gene expression and promote plant resistance to cold stress [47,48]. It has been noted that the cold responsive CBF ERFs (assigned to group IIIc here) contain a series of motifs characterised by clusters of hydrophobic residues delineated by short stretches of acidic residues and prolines within the trans-activating C-terminus [49]. The most highly conserved of these hydrophobic clusters (called HC6 by Wang and colleagues) coincides with the MoRF IIIc C1. Although much of the functional research on the ERFs comes from Arabidopsis, there is also evidence that orthologues from other plants perform functions in similar pathways. For example, overexpression of CBF1 from tomato could also boost cold tolerance in Arabidopsis and mutation of the highly conserved tryptophan residue shown in MoRF IIIc C1 destroyed the trans-activating ability of the tomato orthologue of AtCBF1 [50], highlighting that this MoRF corresponds to a critical motif in these important regulators of plant cold tolerance.

There are two highly conserved MCGGAI(I/L) motif-like MoRFs (Figure 11G, also see VIIc N1* in Supplemental Table S2) that exist in the N-terminal part of the ERFs. In tomato LeERF2 and its homologues from monocot and dicot species are characterized by this unique N-terminal signature. Deletion studies revealed that this motif is required neither for nuclear localization nor for binding to the GCC box, indicating specific functions for the ERFs harbouring the MCGGAI(I/L) signature [51]. This motif is found at the N-terminus of the group VIIa where it appears to be a defining feature that results in these ERFs becoming oxygen and nitric oxide-dependent substrates for the N-end rule targeted degradation [52]. This enables these proteins to act as homeostatic sensors of hypoxia and links them to an ancient and conserved branch of the ubiquitin-mediated proteosomal degradation system. The Met and Cys residues serve as a degron, are specifically cleaved, arginylated (but only in the presence of O or NO) and then recognised by a specific E3 ligase and poly-ubiquitinated. Therefore, it is likely this MoRF is specifically involved in this process.

The Cytokinin Response Factor (CRF) group form yet another distinct subset of ERFs that belong to group VI-L and have been shown to regulate leaf development as part of the cytokinin signal transduction pathway in Arabidopsis. The conserved motifs in the N-terminus of this group of proteins, including one motif that appears to be characteristic of this group that is described as the CRF domain [53]. This corresponds to MoRF VI-L N1 (Supplemental Table S2) that we identified in the disordered *N*-terminus of group VI-L. These ERF proteins affect a set of cytokinin responsive genes that largely overlap with the type-B ARR positive cytokinin regulators and are dependent on the two-component histidine kinase system for cytokinin signal transduction [54]. The CRF domain has been demonstrated to play a key role in interactions between the CRF proteins and with members of the histidine-phosphotransferase component of the cytokinin signalling system [55] and illustrate yet again the importance of MoRFs in ERFs for driving key interactions within the hormone signalling networks that they operate in.

Compared to the 53 conserved motifs identified by sequence alignments in the regions outside of AP2 domains for the 122 Arabidopsis thaliana ERFs [5], our disorder-based potential protein binding sites analyses have resulted in a total 72 conserved MoRFs among the 375 ERFs from three plant species. Among these are 41 conserved MoRFs found in the Arabidopsis thaliana ERFs. This suggests that some conserved motifs generated from sequence alignment may function not by way of modifying direct protein—protein interactions but instead by alternative properties such as maintenance of structural integrity.

### 2.5. Conserved MoRFs are Potentially Mobile Through Evolution

Given the much greater evolutionary distance between mosses and Angiosperms, the moss ERFs are generally distantly related to Arabidopsis and rice ERFs. It can be observed from the conserved MoRFs (Supplemental Table S2) that the moss ERFs frequently have some independently conserved MoRFs themselves. The phylogenetic tree (Figure 1 and Figure 2) also support this tendency with moss ERFs quite often clustering into local clades.

Our analyses suggest the possibility that conserved MoRFs could be shuffled between different protein families during evolution. We have identified a number of cases where conserved MoRFs common in one family or subfamily also occur in a distantly related family or subfamily. These include the C-terminal LWSY-like MoRFs in groups IIa, IIb, IIIb, IIIc, VIIa, VIIIc, and IXa, the C-terminal EDLL-like MoRFs in groups VI, VIIb, IXc and IXd, and the EAR-like MoRFs in groups VI, VIIb, VIIIa, IXc, and IXe (see Supplemental Table S2). Some conserved MoRFs are even found in totally unrelated protein families. For instance, EAR-like MoRFs are also identified in the zinc-finger transcriptional repressors ZAT7, ZAT10, and ZAT11 and play a role in modulating responses to multiple stress factors including drought, cold, pathogens, as well as hormone signalling in general [56]. More recently they were also found in the pathogen effector XopD [57]. The disordered nature of sequences surrounding MoRFs implies that they need not necessarily be highly conserved to retain their disordered structure. This means there is considerable potential for these MoRFs to act as small modules that can be moved around between different proteins by non-homologous recombination events between genes within their respective disordered regions. Where such an event creates a novel functional combination that is beneficial, evolution can then operate to select for the new combination of these modules. This could increase its frequency in a population and sometimes it could then become fixed if the selective advantage is high enough. Given that these types of events provide a method for coordinating protein–protein interaction networks and thereby can initiate cross-talk and extra robustness in networks, the potential for significant evolutionary advantages gained by such a route is substantial. We surmise that this may well be one of the forces operating on re-distribution of conserved MoRFs between related (and occasionally even unrelated) protein families that display disorder. An extreme case of this may be the presence of two EAR-like MoRFs in the pathogen effector XopD which are required to target the tomato ERF SlERF4 for desumoylation and suppress the ethylene response [57]. These may be acting as a mimic of the host EAR-like MoRFs present in ERF groups VI, VIIb, VIIIa, IXc, and IXe. While a horizontal gene transfer event between the host and pathogen is a possible origin for this novel effector construct, given that the length of some of these MoRFs is short some could alternatively evolve by convergent evolution pathways.

## 3. Materials and Methods

### 3.1. Sequence and Phylogenetic Analysis

All ERF sequences of three diverse species were selected for this study. This includes 122 Arabidopsis (*Arabidopsis thaliana*) EFRs from TAIR, 133 rice ERFs (*Oryza sativa* L. subsp. *japonica*) from PlantTFDB (http://planttfdb.cbi.pku.edu.cn/index.php) and 120 Moss ERFs (*Physcomitrella patens*) from iTAK (http://bioinfo.bti.cornell.edu/cgi-bin/itak/index.cgi). Some individual ERFs with unusual features such as double or incomplete AP2 domains are excluded from this analysis. All sequence alignments were carried out using Clustal Omega (http://www.ebi.ac.uk/Tools/msa/clustalo/) with manual adjustment in some alignments by placing a greater emphasis on motifs harbouring MoRFs. A phylogenetic tree was constructed using the neighbour-joining method with bootstrapping (1000) in Geneious.

### 3.2. Amino Acids Compositional Profile Analysis

The relative compositional profile is defined by (C_i_-C_i_^FSP^)/C_i_^FSP^, where C_i_ is the absolute compositional percentage of ith amino acid in the query dataset and C_i_^FSP^ is the absolute compositional percentage of ith amino acid in the fully-structured protein dataset (FSP) [58]. The FSP is composed of sequences which are monomeric, non-membrane protein crystal structures extracted from Protein Data Bank. The FSP candidate sequences with 25% or higher sequence identity were filtered to keep the longest one, and the final FSP dataset has 554 protein sequences and 113,895 residues.

### 3.3. Charge-Hydropathy (CH) and Cumulative Distribution Function (CDF) Plot

ERF sequences were subjected to a combined analysis of Charge-Hydropathy (CH) plot [18] and Cumulative Distribution Function (CDF) plot [59]. CH plot consists of plotting the averaged Kyte-Doolittle hydrophobicity of a protein on the X-axis against the averaged net charges of the same protein on the Y-axis. There is a boundary line that divides the CH plot into the upper-left region where disordered proteins are normally located and the lower-right region where structured proteins are normally located. CDF plot is a cumulated histogram of disordered residues with various disordered score. Similar to CH plots there is also a boundary line identified in the CDF plot to separate disordered proteins from structured proteins. The disordered scores and CDF distance used for CDF plot are from the PONDR-VSL2 disorder predictor [20]. The distances to the boundary lines in both CDF and CH plots from a specific protein were further used as coordinates on x- and y-axes to develop a combined CH-CDF plot.

### 3.4. Disorder Predictions

Protein disorder predictions were carried out using predictor PONDR-FIT which is one of the predictors with high prediction accuracy [60]. Disorder curves were aligned using IDAlign (IDAA; [61]). Molecular Recognition Features (MoRFs) were identified using MoRFchibi predictors (http://morf.chibi.ubc.ca:8080/mcw/index.html; [62]) and were also plotted onto the PONDR-FIT profiles. Sequence complexity analyses were carried out by using an iterative algorithm (CAST; http://athina.biol.uoa.gr/CAST/; [24]). The fraction of intrinsically disordered amino acids (IDAA%) is the percentage of predicted disorder (promoting) amino acids in a certain length of sequence.

### 3.5. Predictions of Phosphorylation Sites

The predictions of phosphorylated sites are carried out by using the Musite predictor which integrates both protein disorder and amino acid specificity. Musite is a bioinformatics tool for predicting both general and kinase-specific protein phosphorylation sites. (http://musite.net/; [63]).

## 4. Conclusions

MoRFs are short interaction-prone fragments located within extended disorder regions and can undergo disorder-to-order transitions upon binding to their interacting partners. We suggest the MoRFs we have identified in ERFs use these properties to act in complex networks. Several of the conserved MoRFs identified here are verified by overlapping with experimentally confirmed binding sites including the AP2 DNA binding sites and other motifs outside of AP2 domains referred to above. Given the disordered nature of the TRDs, it is likely that many of the protein interactions between ERFs and their partners follow the disorder-to-order transition mode in response to signal stimuli. In this way they can regulate complicated interaction networks that can be turned on, off, or maintained in a dynamic state of equilibrium. One example of this is the EDLL-like activation MoRF that can overcome the repression of the EAR-like MoRF under some circumstances [46]. Our analysis suggests conserved MoRFs should become focal points for functional studies of each ERF subgroup. The AP2 domain-associated components can lead to divergent DNA binding specificities and, combined with the likely disorder/flexibility-based interactions of the MoRFs in the N- and C- terminal disordered domains, can reasonably explain the versatility of regulatory roles played by this important plant protein family. We suggest therefore that disorder in ERFs has played a crucial role, preparing sessile plants for the multitude of stresses that they will inevitably face in their lifetime and allowing flowering plants to adapt to a rich variety of biological niches.

## Figures and Tables

**Table 1:** Fraction of predicted phosphorylation of 375 ERFs by residue types and domains.

	S	T	Y	All (S + T + Y)
AP2 domain only	0.28%	0.10%	34.29%	11.15%
Non-AP2 domain	8.59%	5.93%	24.85%	9.67%
Full-length	8.09%	4.92%	30.32%	9.97%

## Supplementary Materials

Supplementary materials can be found at https://www.mdpi.com/1422-0067/21/1/74/s1. Table S1. New and previous grouping of ERFs (where applicable) along with their alternative names. Table S2. Summary of conserved MoRFs in carboxy and amino terminal regions outside of the AP2 domains. Figure S1. Phylogenetic trees of a representative set of groups/subgroups. Figure S2. Disorder prediction of ERFs. Figure S3. Alignments of some ERF subgroups. File S1. Table S1 References.
