# Supplementary material for: Computational Disorder Analysis in Ethylene Response Factors Uncovers Binding Motifs Critical to Their Diverse Functions
Source: Int J Mol Sci. 2019 Dec 20;21(1):74. doi: 10.3390/ijms21010074 (PMC6981732; doi:10.3390/ijms21010074)
Supplement: Supplementary file 1 [file ijms-21-00074-s001.zip › ijms-650542-supplementary-XML/Figure S2.pdf]

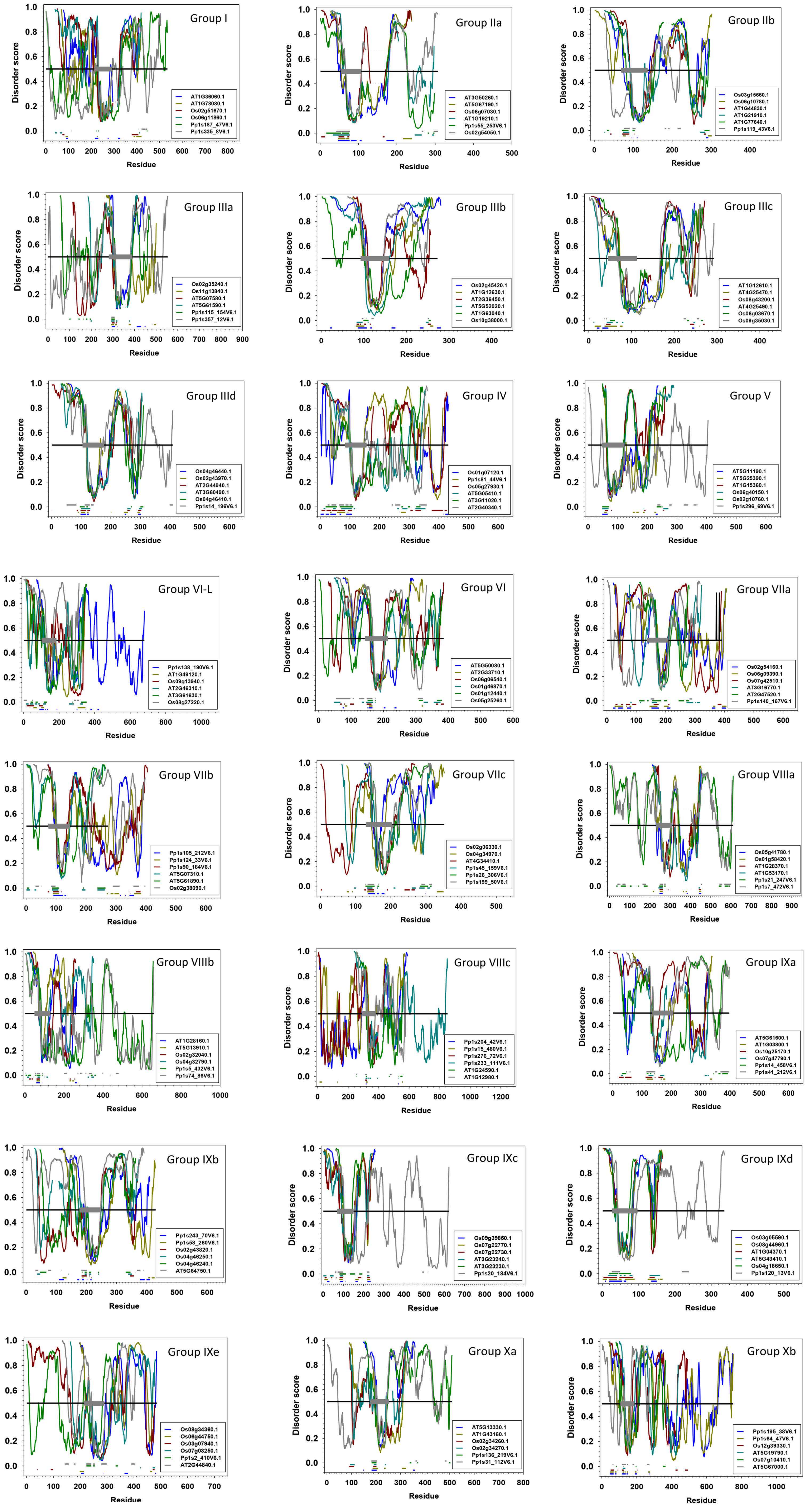

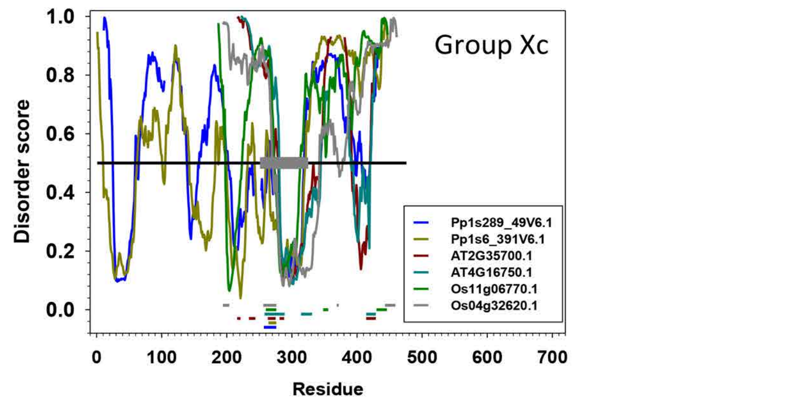

Supplemental Figure S2: Disorder prediction of ERFs. All of the subgroups are presented herein. Disorder score was predicted using PONDR-FIT. The disorder curves were aligned using IDAlign. The grey thick bars indicate the span of the AP2 (DBD) domains including the three highly structured beta sheet and the more flexible alpha helices at the C-terminal end of this domain. The black horizontal line represents the disorder threshold score (⩾ 0.5). The short horizontal color bars at the bottom of each panel are MoRFs predicted using MoRFchibi.
