# Supplementary material for: Computational Disorder Analysis in Ethylene Response Factors Uncovers Binding Motifs Critical to Their Diverse Functions
Source: Int J Mol Sci. 2019 Dec 20;21(1):74. doi: 10.3390/ijms21010074 (PMC6981732; doi:10.3390/ijms21010074)
Supplement: Supplementary file 1 [file ijms-21-00074-s001.zip › ijms-650542-supplementary-XML/Figure S3.pdf]

Group I

```
1. Os01g66270.1      RYRGVRRRRWGKWVSEIRVPGTRERLWLGSYATPEAAAVAHDTAVYFLRGGAG--DGGGGATLNFP
2. Os05g34730.1      RYRGVRRRRWGKWVSEIRVPGTRERLWLGSYATAEAAAVAHDAAVCLLRLGGGRRAAAGGGGGLNFP
3. AT1G22190.1       LYRGVRQRHWGKWVAEIRLPRNRTRLWLGTFDTAEEAALAYDKAAYKLR----------GDFARLNFP
4. AT1G78080.1       LYRGVRQRHWGKWVAEIRLPRNRTRLWLGTFDTAEEAALAYDKAAYKLR----------GDFARLNFP
5. AT1G36060.1       LYRGVRQRHWGKWVAEIRLPRNRTRLWLGTFDTAEEAALAYDRAAFKLR----------GDSARLNFP
6. Os03g09170.1      LYRGVRQRHWGKWVAEIRLPRNRTRLWLGTFDTAEEAALAYDSAAFRLR----------GESARLNFP
7. Os10g22600.1      LYRGVRQRHWGKWVAEIRLPRNRTRLWLGTYDTAEDAALAYDGAAFRLR----------GDAARLNFP
8. Os09g20350.1      LYRGVRQRHWGKWVAEIRLPRNRTRLWLGTFDTAEEAALAYDQAAYRLR----------GDAARLNFP
9. Os08g31580.1      LYRGVRQRHWGKWVAEIRLPRNRTRLWLGTFDTAEEAALTYDQAAYRLR----------GDAARLNFP
10. Os02g51670.1     LYRGVRQRHWGKWVAEIRLPKNRTRLWLGTFDTAEDAALAYDKAAFRLR----------GDLARLNFP
11. Os06g11860.1     MYRGVRQRHWGKWVAEIRLPKNRTRLWLGTFDTAEDAALAYDKAAFRLR----------GDAARLNFP
12. AT2G22200.1      LYRGVRQRHWGKWVAEIRLPKNRTRLWLGTFETAEKAALAYDQAAFQLR----------GDIAKLNFP
13. AT4G39780.1      LYRGVRQRHWGKWVAEIRLPKNRTRLWLGTFDTAEEAAMAYDLAAYKLR----------GEFARLNFP
14. AT5G65130.1      LYRGVRQRQWGKWVAEIRLPKNRTRLWLGTFETAQEAALAYDQAAHKIR----------GDNARLNFP
15. Pp1s187_47V6.1   LYRGVRQRHWGKWVAEIRLPRNRTRLWLGTFDTAEEAALAYDAAAYKLR----------GEYARLNFP
16. Pp1s335_8V6.1    LYRGVRQRHWGKWVAEIRLPRNRTRLWLGTFETAEEAAMAYDAAAYKLR----------GEYARLNFP
17. Pp1s21_214V6.1   KYRGVRQRHWGSWVSEIRHPQLKTRVWLGTFETAEDAAHAYDEAARLMC----------GAKARTNFP
18. Pp1s79_25V6.1    RYRGVRQRHWGSWVSEIRHPLLKTRVWLGTFETAEDAAHAYDEAARLMC----------GARARTNFP
19. AT1G64380.1      LYRGVRQRHWGKWVAEIRLPQNRMRVWLGTYDTAEAAAYAYDRAAYKLR----------GEYARLNFP
20. Os04g44670.1     LYRGVRQRHWGKWVAEIRLPRNRTRLWLGTFDTAEDAAMAYDREAFKLR----------GENARLNFP
```

Group IIb

```
1. AT1G21910.1       YKGVRMR-SWGSWVSEIRAPN-QKTRIWLGSYSTAEAAARAYDVALLCLKGPQAN-LNF
2. AT1G77640.1       YKGVRMR-SWGSWVTEIRAPN-QKTRIWLGSYSTAEAAARAYDAALLCLKGPKAN-LNF
3. AT1G44830.1       YKGVRMR-SWGSWVSEIRAPN-QKTRIWLGSYSTAEAAARAYDAALLCLKGSSANNLNF
4. Os06g10780.1      YKGVRMR-SWGSWVSEIRAPS-QKTRIWLGSYSTAEAAARAYDAALLCLKGSAAADLNF
5. Os03g15660.1      YKGVRMR-SWGSWVSEIRAPH-QKRRIWLGSYATPEAAARAYDAALLCLKGSDAV-LNF
6. Pp1s119_43V6.1    FKGIRYRPELGRYISEIRPAQPRKRKIWLGTFKTAEEAARAFDAGIFYTKKPVEY--NF
7. Pp1s86_33V6.1     FKGIRFRPELGRYISEIRPAHPRKRKIWLGTFKTAEEAARAFDAGIFYTKKPIDY--NF
8. Pp1s11_280V6.1    YKGIRYRPELGRYISEIRPAQPRKRKIWLGTYKTAEEAARAFDAGIFYTKKPINY--NF
9. Pp1s87_98V6.1     YKGIRYRPELNRYISEIRPVQPRKRKIWLGTYKTPEEAARAFDAGIFYTKKQIDY--NF
10. AT4G31060.1      YRGVRKR-SWGKWVSEIRVPK-TGRRIWLGSYDAPEKAARAYDAALFCIRGEKGV-YNF
11. Pp1s373_18V6.1   YRGVRHRPELNKWVTEIRPTA-QKRKIWLGTYKTAEEAARAYDVGIFYTGKKIPL--NF
```

Group IIIc

```
1. Os09g35020.1      KRPAGRTKFRETRHPVYRGVRRRGG----AGRWVCEVRVPGKRGARLWLGTYVTAEAAARAHDAAMIALR-GGAGGGGAACLNF
2. Os09g35030.1      KRPAGRTKFRETRHPVFRGVRRRGN----AGRWVCEVRVPGRRGCRLWLGTFDTAEGAARAHDAAMLAINAGGGGGGGACCLNF
3. Os09g35010.1      KRPAGRTKFRETRHPVYRGVRRRGGRPGAAGRWVCEVRVPGARGSRLWLGTFATAEAAARAHDAAALALR------GRAACLNF
4. Os08g43200.1      KRPAGRTKFQETRHPVFRGVRRRGR----AGRWVCEVRVPGSRGDRLWVGTFDTAEEAARAHDAAMLALC------GASASLNF
5. Os08g43210.1      KRPAGRTKFHETRHPVFRGVRRRGR----AGRWVCEVRVPGRRGCRLWLGTFDAADAAARAHDAAMLALR-----GRAAACLNF
6. Os06g03670.1      KRPAGRTKFRETRHPVYRGVRRRGP----AGRWVCEVREPNKK-SRIWLGTFATAEAAARAHDVAALALR------GRGACLNF
7. AT1G12610.1       KKRAGRRVFKETRHPVYRGIRRRNG-----DKWVCEVREPTHQ-RRIWLGTYPTADMAARAHDVAVLALR------GRSACLNF
8. AT1G63030.1       KKRAGRRIFKETRHPIYRGVRRRDG-----DKWVCEVREPIHQ-RRVWLGTYPTADMAARAHDVAVLALR------GRSACLNF
9. AT4G25470.1       KKPAGRKKFRETRHPIYRGVRQRNS-----GKWVCELREPNKK-TRIWLGTFQTAEMAARAHDVAAIALR------GRSACLNF
10. AT4G25490.1      KKPAGRKKFRETRHPIYRGVRQRNS-----GKWVSEVREPNKK-TRIWLGTFQTAEMAARAHDVAALALR------GRSACLNF
11. AT4G25480.1      KKPAGRKKFRETRHPIYRGVRRRNS-----GKWVCEVREPNKK-TRIWLGTFQTAEMAARAHDVAALALR------GRSACLNF
12. AT5G51990.1      KKRAGRKKFRETRHPIYRGVRQRNS-----GKWVCEVREPNKK-SRIWLGTFPTVEMAARAHDVAALALR------GRSACLNF
13. Os02g45450.1     KRRAGRTKFKETRHPVFKGVRRRNP-----GRWVCEVREPHGK-QRIWLGTFETAEMAARAHDVAALALR------GRAACLNF
14. Os04g48350.1     KRRAGRTKFKETRHPVYKGVRSRNP-----GRWVCEVREPHGK-QRIWLGTFETAEMAARAHDVAAMALR------GRAACLNF
15. Os01g73770.1     KRRAGRKKFRETRHPVYRGVRARAG----GSRWVCEVREPQAQ-ARIWLGTYPTPEMAARAHDVAAIALR-----GERGAELNF
```

Group VIIa

```
1. AT1G72360.1       YKGIRRRPWGRWAAEIRDP-IKGVRVWLGTFNTAEEAARAYDLEAKRIRGAKAKLNFP
2. Os07g42510.1      YRGIRQRPWGKWAAEIRDP-RKGVRVWLGTFGTAEEAAMAYDVEARRIRGKKAKVNFP
3. AT2G47520.1       YRGIRQRPWGKWAAEIRDP-SKGVRVWLGTFKTADEAARAYDVAAIKIRGRKAKLNFP
4. Os03g08470.1      FRGIRQRPWGKWAAEIRDP-HKGTRVWLGTFNTPEEAARAYDVEARRLRGSKAKVNFP
5. Os02g54160.1      FRGIRQRPWGKWAAEIRDP-RKGVRVWLGTFNSAEEAARAYDAEARRIRGKKAKVNFP
6. Os06g09390.1      FRGIRQRPWGKWAAEIRDP-RKGVRVWLGTFNSPEEAARAYDAEARRIRGKKAKVNFP
7. Os09g26420.1      YRGIRQRPWGKWAAEIRDP-SKGVRVWLGTYNTAEEAARAYDAEARKIRGKKAKVNFP
8. AT1G53910.1       YRGIRQRPWGKWAAEIRDP-REGARIWLGTFKTAEEAARAYDAAARRIRGSKAKVNFP
9. AT3G14230.1       YRGIRQRPWGKWAAEIRDP-RKGSREWLGTFDTAEEAARAYDAAARRIRGTKAKVNFP
10. AT3G16770.1      YRGIRKRPWGKWAAEIRDP-RKGVRVWLGTFNTAEEAAMAYDVAAKQIRGDKAKLNFP
11. Os05g29810.1     YRGIRRRPWGRWAAEIRDP-RKGARVWLGTYATAEEAARAYDVAARDIRGAKAKLNFP
12. Os01g54890.1     YRGVRRRPWGKYAAEIRDPTRHGARLWLGTFGTAEEAAAAYDRAAFRMRGAKALLNFP
13. Pp1s140_167V6.1  FRGGRQRPWGKFAAEIRDSTRHGSRLWLGTFDTAEEAALACDDAAIQLRGSRALLNFP
```
