# Supplementary material for: Computational Disorder Analysis in Ethylene Response Factors Uncovers Binding Motifs Critical to Their Diverse Functions
Source: Int J Mol Sci. 2019 Dec 20;21(1):74. doi: 10.3390/ijms21010074 (PMC6981732; doi:10.3390/ijms21010074)
Supplement: Supplementary file 1 [file ijms-21-00074-s001.zip › ijms-650542-supplementary-XML/Supplemental File Legends.pdf]

Supplemental Table S1: New and previous grouping of ERFs (where applicable) along with their alternative names. The stress function and/or hormonal pathways that representative genes in the group have been linked with (noted in red) are given and represented pictorially in Figure 1. More distantly related genes that have uncertain group membership status are labelled ①. On the phylogenetic trees they occupy a position that is basal to the rest of the members of the group. They have also been tested for membership of other groups but appear not to fit with any other group. † Grouping of this ERF by Nakano et al. (2006) where it exists while its grouping by Sakuma et al. (2002) where it exists is given in brackets; ‡ Functions associated with a particular hormone and/or the type of stress. Superscripted numbers refer to source references (see Supplemental File S1) for the information linking the gene to the stipulated stress, hormone or function.

Supplemental Table S2: Summary of conserved MoRFs. Summary of conserved MoRFs in carboxy and amino terminal regions outside of the AP2 domains. The numbers in front represent groups/subgroups, followed by N (prior to the AP2 domain) or C (after the AP2 domain). The asterisks indicate MoRFs exclusive to the group.

Supplemental File S1: References cited in Supplemental Table S1.

Supplemental Figure S1: Phylogenetic trees of a representative set of groups/subgroups. Subgroup phylogenetic trees to illustrate how the groupings were tested and genes positioned basally to all other members of a group were identified for potential re-assignment to other groups/subgroups. Pp1s107_32V6.1 was used as the outgroup. For example, in group I gene Os04g44670.1 was identified as an outlier and retested, did not fit with any other group/subgroup and was therefore retained in group I.

Supplemental Figure S2: Disorder prediction of ERFs. All of the subgroups are presented herein. Disorder score was predicted using PONDR-FIT. The disorder curves were aligned using IDAlign. The grey thick bars indicate the span of the AP2 (DBD) domains including the three highly structured beta sheet and the more flexible alpha helices at the C-terminal end of this domain. The black horizontal line represents the disorder threshold score (≥ 0.5). The short horizontal color bars at the bottom of each panel are MoRFs predicted using MoRFchibi.

Supplemental Figure S3: Alignments of some ERF subgroups. Examples of alignments of the AP2/ERF domain in four subgroups of ERF proteins.
