# Supplementary material for: Computational Disorder Analysis in Ethylene Response Factors Uncovers Binding Motifs Critical to Their Diverse Functions
Source: Int J Mol Sci. 2019 Dec 20;21(1):74. doi: 10.3390/ijms21010074 (PMC6981732; doi:10.3390/ijms21010074)
Supplement: Supplementary file 1 [file ijms-21-00074-s001.zip › ijms-650542-supplementary-XML/Supplementary File S1.pdf]

Supplementary File S1

Disorder analysis in ERFs identifies functional binding motifs; Plant Molecular Biology; Xiaolin Sun, Nawar Malhis, Bi Zhao, Bin Xue, Joerg Gsponer and Erik H.A. Rikkerink; Corresponding author Erik H.A. Rikkerink; The New Zealand Institute for Plant & Food Research Ltd;
erik.rikkerink@plantandfood.co.nz

Table 1 References

1. Abogadallah GM, Nada RM, Malinowski R, Quick P. 2011. Overexpression of HARDY, an AP2/ERF gene from Arabidopsis, improves drought and salt tolerance by reducing transpiration and sodium uptake in transgenic *Trifolium alexandrinum L.* Planta 233, 1265-1276.

2. Ali MA, Abbas A, Kreil DP, Bohlmann H. 2013. Overexpression of the transcription factor RAP2.6 leads to enhanced callose deposition in syncytia and enhanced resistance against the beet cyst nematode Heterodera schachtii in Arabidopsis roots. BMC Plant Biology 13, 47.

3. Berrocal-Lobo M, Molina A, Solano R. 2002. Constitutive expression of ETHYLENE-RESPONSE-FACTOR1 in Arabidopsis confers resistance to several necrotrophic fungi. Plant Journal 29, 23-32.

4. Bolt S, Zuther E, Zintl S, Hincha DK, Schmulling T. 2017. ERF105 is a transcription factor gene of Arabidopsis thaliana required for freezing tolerance and cold acclimation. Plant Cell and Environment 40, 108-120.

5. Brodersen P Petersen M, Nielsen HB, Zhu S, Newman M-A, Shokat KM, Rietz S, Parker J, Mundy J. 2006. Arabidopsis MAP kinase 4 regulates salicylic acid- and jasmonic acid/ethylene-dependent responses via EDS1 and PAD4. Plant Journal 47, 532-546.

6. Cao YF, Song FM, Goodman RM, Zheng Z. 2006. Molecular characterization of four rice genes encoding ethylene-responsive transcriptional factors and their expressions in response to biotic and abiotic stress. Journal of Plant Physiology 163, 1167-1178.

7. Chandler JW. 2018. Class VIIIb APETALA2 Ethylene Response Factors in Plant Development. Trends in Plant Science 23, 151-162.

8. Chen WH, Li PF, Chen MK, Lee YI, Yang CH. 2015. FOREVER YOUNG FLOWER Negatively Regulates Ethylene Response DNA-binding Factors (EDFs), by Activating an Ethylene-Responsive Factor (ERF), to Control Arabidopsis Floral Organ Senescence and Abscission. Plant Physiology 168, 1666–1683.

9. Chilley PM, Casson SA, Tarkowski P, Hawkins N, Wang Kevin L-C, Hussey Patrick J, Beale M, Ecker JR, Sandberg GK, Lindsey K. 2006. The POLARIS Peptide of Arabidopsis Regulates Auxin Transport and Root Growth via Effects on Ethylene Signaling. Plant Cell 18, 3058-3072.

10. Choi SW, Lee SB, Na YJ, Jeung SG, Kim SY. 2017. Arabidopsis MAP3K16 and Other Salt-Inducible MAP3Ks Regulate ABA Response Redundantly. Molecules and Cells 40, 230-242.

11. Cole, Melanie, Chandler, John, Weijers, Dolf, Jacobs, Bianca, Comelli, Petra, Werr, Wolfgang. 2009. DORNROSCHEN is a direct target of the auxin response factor MONOPTEROS in the Arabidopsis embryo. Development 136, 1643-1651

12. Cui M, Zhang WJ, Zhang Q, Xu ZQ, Zhu ZG, Duan FP, Wu R. 2011. Induced over-expression of the transcription factor OsDREB2A improves drought tolerance in rice. Plant Physiology and Biochemistry 49, 1384-1391.

13. Cutliffe JW, Hellmann E, Heyl A, Rashotte AM 2011. CRFs form protein-protein interactions with each other and with members of the cytokinin signalling pathway in Arabidopsis via the CRF domain. Journal of Experimental Botany 62, 4995-5002.

14. Dong CJ, Liu JY. 2010. The Arabidopsis EAR-motif-containing protein RAP2.1 functions as an active transcriptional repressor to keep stress responses under tight control. BMC Plant Biology 10, 15.

15. Dubouzet JG, Sakuma Y, Ito Y, Kasuga M, Dubouzet EG, Miura S, Seki M, Shinozaki K, Yamaguchi-Shinozaki K. 2003. OsDREB genes in rice, *Oryza sativa L.*, encode transcription activators that function in drought-, high-salt- and cold-responsive gene expression. Plant Journal 33, 751-763.

16. Duran-Medina Y, Serwatowska J, Reyes-Olalde JI, de Folter S, Marsch-Martinez N. 2017. The AP2/ERF Transcription Factor DRNL Modulates Gynoecium Development and Affects Its Response to Cytokinin. Frontiers in Plant Science 8.

17. Figueiredo DD, Barros PM, Cordeiro AM, Serra TS, Lourenco T, Chander S, Oliveira MM, Saibo NJM. 2012. Seven zinc-finger transcription factors are novel regulators of the stress responsive gene OsDREB1B. Journal of Experimental Botany 63, 3643-3656.

18. Guo R, Sun W. 2017. Sumoylation stabilizes RACK1B and enhance its interaction with RAP2.6 in the abscisic acid response. Scientific Reports 7, 44090.

19. Guo Y, Huang R, Duan L, Wang J. 2017. The APETALA2/ethylene-responsive factor transcription factor OsDERF2 negatively modulates drought stress in rice by repressing abscisic acid responsive genes. Journal of Agricultural Science 155, 966-977.

20. Haake V, Cook D, Riechmann JL, Pineda O, Thomashow MF, Zhang JZ. 2002. Transcription factor CBF4 is a regulator of drought adaptation in Arabidopsis. Plant Physiology 130, 639-648.

21. Hsieh EJ, Cheng MC, Lin TP. 2013. Functional characterization of an abiotic stress-inducible transcription factor AtERF53 in *Arabidopsis thaliana*. Plant Molecular Biology 82, 223-237.

22. Hu YB, Zhao LF, Chong K, Wang T. 2008. Overexpression of OsERF1, a novel rice ERF gene, up-regulates ethylene-responsive genes expression besides affects growth and development in Arabidopsis. Journal of Plant Physiology 165, 1717-1725.

23. Huibers RP, de Jong M, Dekter RW, Van der Ackerveken G. 2009. Disease-Specific Expression of Host Genes During Downy Mildew Infection of Arabidopsis. Molecular Plant-Microbe Interactions 22, 1104-1115.

24. Hwang JE, Lim CJ, Chen H, Je J, Song C, Lim CO. 2012. Overexpression of Arabidopsis Dehydration-Responsive Element-Binding Protein 2C Confers Tolerance to Oxidative Stress. Molecules and Cells 33, 135-140.

25. Ito Y, Katsura K, Maruyama K, Taji T, Kobayashi M, Seki M, Shinozaki K, Yamaguchi-Shinozaki K. 2006. Functional analysis of rice DREB1/CBF-type transcription factors involved in cold-responsive gene expression in transgenic rice. Plant and Cell Physiology 47, 141-153.

26. Iwase A, Ohme-Takagi M, Sugimoto K. 2011. WIND1: A key molecular switch for plant cell dedifferentiation. Plant Signaling & Behavior 6, 1943-1945.

27. Je J, Chen H, Song C, Lim CO. 2014. Arabidopsis DREB2C modulates ABA biosynthesis during germination. Biochemical and Biophysical Research Communications 452, 91-98.

28. Joo J, Choi HJ, Lee YH, Kim YK, Song SI. 2013. A transcriptional repressor of the ERF family confers drought tolerance to rice and regulates genes preferentially located on chromosome 11. Planta 238, 155-170.

29. Jung H, Chung PJ, Park SH, Redillas M, Kim YS, Suh JW, Kim JK. 2017. Overexpression of OsERF48 causes regulation of OsCML16, a calmodulin-like protein gene that enhances root growth and drought tolerance. Plant Biotechnology Journal 15, 1295-1308.

30. Kang, H. G., Kim, J., Kim, B., Jeong, H., Choi, S. H., Kim, E. K., Lee, H. Y., Lim, P. O. 2011. Overexpression of FTL1/DDF1, an AP2 transcription factor, enhances tolerance to cold, drought, and heat stresses in *Arabidopsis thaliana*. Plant Science 180, 634-641.

31. Karaba A, Dixit S, Greco R, Aharoni A, Trijatmiko KR, Marsch-Martinez N, Krishnan A, Nataraja KN, Udayakumar M, Pereira A. Improvement of water use efficiency in rice by expression of HARDY, an Arabidopsis drought and salt tolerance gene. Proceedings of the National Academy Of Sciences USA 104, 15270-15275.

32. Kim, M. J., Ruzicka, D., Shin, R., Schachtman, D. P. 2012. The Arabidopsis AP2/ERF Transcription Factor RAP2.11 Modulates Plant Response to Low-Potassium Conditions. Molecular Plant 5, 1042-1057.

33. Komatsu M, Chujo A, Nagato Y, Shimamoto K, Kyozuka J. 2003. FRIZZY PANICLE is required to prevent the formation of axillary meristems and to establish floral meristem identity in rice spikelets. Development 130, 3841-3850.

34. Lasserre E, Jobet E, Llauro C, Delseny M. 2008. AtERF38 (At2g35700), an AP2/ERF family transcription factor gene from *Arabidopsis thaliana*, is expressed in specific cell types of roots, stems and seeds that undergo suberization. Plant Physiology and Biochemistry 46, 1051-1061.

35. Lee DK, Jung H, Jang G, Jeong JS, Kim YS, Ha SH, Do Choi Y, Kim JK. 2016. Overexpression of the OsERF71 Transcription Factor Alters Rice Root Structure and Drought Resistance. Plant Physiology 172, 575-588.

36. Lee SB, Lee SJ, Kim SY. 2015. AtERF15 is a positive regulator of ABA response. Plant Cell Reports 34, 71-81.

37. Lee SJ, Park JH, Lee MH, Yu JH, Kim SY. 2010. Isolation and functional characterization of CE1 binding proteins. BMC Plant Biology 10, 277

38. Li ZF, Zhang LX, Yu YW, Quan RD, Zhang ZJ, Zhang HW, Huang RF. 2011. The ethylene response factor AtERF11 that is transcriptionally modulated by the bZIP transcription factor HY5 is a crucial repressor for ethylene biosynthesis in Arabidopsis. Plant Journal 68, 88-99.

39. Lim CJ, Hwang JE, Chen H, Hong JK, Yang KA, Choi MS, Lee KO, Chung WS, Lee SY, Lim CO. 2007. Over-expression of the Arabidopsis DRE/CRT-binding transcription factor DREB2C enhances thermotolerance. Biochemical and Biophysical Research Communications 362, 431-436.

40. Lin R, Zhao W, Meng X, Peng YL. 2007. Molecular cloning and characterization of a rice gene encoding AP2/EREBP-type transcription factor and its expression in response to infection with blast fungus and abiotic stresses. Physiological and Molecular Plant Pathology 70, 60-68.

41. Liu DF, Chen XJ, Liu JQ, Ye JC, Guo ZJ. 2012. The rice ERF transcription factor OsERF922 negatively regulates resistance to *Magnaporthe oryzae* and salt tolerance. Journal of Experimental Botany 63, 3899-3911.

42. McGrath KC, Dombrecht B, Manners JM, Schenk PM, Edgar CI, Maclean DJ, Scheible WR, Udvardi MK, Kazan K 2005. Repressor- and activator-type ethylene response factors functioning in jasmonate signaling and disease resistance identified via a genome-wide screen of Arabidopsis transcription factor gene expression. Plant Physiology 139, 949-959.

43. Mao JL, Miao ZQ, Wang Z, Yu LH, Cai XT, Xiang CB. 2016. Arabidopsis ERF1 Mediates Cross-Talk between Ethylene and Auxin Biosynthesis during Primary Root Elongation by Regulating ASA1 Expression. Plos Genetics 12.

44. Maruyama Y, Yamoto N, Suzuki Y, Chiba Y, Yamazaki K, Sato T, Yamaguchi J. 2013 The Arabidopsis transcriptional repressor ERF9 participates in resistance against necrotrophic fungi. Plant Science 213, 79-87.

45. Nakashima K, Shinwari ZK, Sakuma Y, Seki M, Miura S, Shinozaki K, Yamaguchi-Shinozaki K. 2000. Organization and expression of two Arabidopsis DREB2 genes encoding DRE-binding proteins involved in dehydration- and high-salinity-responsive gene expression. Plant Molecular Biology 42, 657-665.

46. Pandey GK, Grant JJ, Cheong YH, Kim BG, Li L, Luan S. 2005. ABR1, an APETALA2-domain transcription factor that functions as a repressor of ABA response in Arabidopsis. Plant Physiology 139, 1185-1193.

47. Papdi C, Perez-Salamo I, Joseph MP, Giuntoli B, Bogre L, Koncz C, Szabados L. 2015. The low oxygen, oxidative and osmotic stress responses synergistically act through the ethylene response factor VII genes RAP2.12, RAP2.2 and RAP2.3. Plant Journal 82, 772-784.

48. Pré M, Atallah M, Champion A, De Vos M, Pieterse CMJ, Memelink J, 2008. The AP2/ERF domain transcription factor ORA59 integrates jasmonic acid and ethylene signals in plant defense. Plant Physiology 147, 1347-1357.

49. Raines T, Shanks C, Cheng CY, McPherson D, Argueso CT, Kim HJ, Franco-Zorrilla JM, Lopez-Vidriero I, Solano R, Vankova R, Schaller GE, Kieber JJ. 2016. The cytokinin response factors modulate root and shoot growth and promote leaf senescence in Arabidopsis. Plant Journal 85, 134-147.

50. Ramaiah M, Jain A, Raghothama KG. 2014. AtERF070 Regulates Root Development and Phosphate-Starvation-Mediated-Responses. Plant Physiology 164, 1484-98

51. Sakuma Y, Liu Q,, Dubouzet JG, Abe H, Shinozaki K, Yamaguchi-Shinozaki K. 2002. DNA-Binding Specificity of the ERF/AP2 Domain of Arabidopsis DREBs, Transcription Factors Involved in Dehydration- and Cold-Inducible Gene Expression. Biochemical and Biophysical Research Communications 290, 998–1009

52. Scarpeci TE, Frea VS, Zanor MI, Valle EM. 2017. Overexpression of AtERF019 delays plant growth and senescence, and improves drought tolerance in Arabidopsis. Journal of Experimental Botany 68, 673-685.

53. Schaller GE, Voesenek L. 2015. Focus on Ethylene. Plant Physiology 169, 1-2.

54. Schmidt R, Mieulet D, Hubberten HM, Obata T, Hoefgen R, Fernie AR, Fisahn J, Segundo BS, Guiderdoni E, Schippers JHM, Mueller-Roeber B. 2013. SALT-RESPONSIVE ERF1 Regulates Reactive Oxygen Species-Dependent Signaling during the Initial Response to Salt Stress in Rice. Plant Cell 25, 2115-2131.

55. Seo YS, Chern M, Bartley LE, Han M, Jung K-H, Lee I, Walia H, Richter T, Xu X, Cao P, Bai W, Ramanan R, Amonpant F, Arul L, Canlas PE, Ruan R, Park C-J, Chen X, Hwang S, Jeon J-S, Ronald PC. 2011. Towards establishment of a rice stress response Interactome. PLoS Genetics 7, e1002020.

56. Serra TS, Figueiredo DD, Cordeiro AM, Almeida DM, Lourenco T, Abreu IA, Sebastian A, Fernandes L, Contreras-Moreira B, Oliveira MM, Saibo NJM. 2013. OsRMC, a negative regulator of salt stress response in rice, is regulated by two AP2/ERF transcription factors. Plant Molecular Biology 82, 439-455.

57. Shu K, Zhou W, Yang W. 2018. APETALA 2-domain-containing transcription factors: focusing on abscisic acid and gibberellins antagonism. New Phytologist 217, 977-983.

58. Song C, Je J, Hong JK, Lim CO. 2014. Ectopic expression of an Arabidopsis dehydration-responsive element-binding factor DREB2C improves salt stress tolerance in crucifers. Plant Cell Reports 33, 1239-1254.

59. Song C-P, Galbraith DW. 2006 AtSAP18, an orthologue of human SAP18, is involved in the regulation of salt stress and mediates transcriptional repression in Arabidopsis. Plant Molecular Biology 60, 241-257.

60. Sun S, Yu JP, Chen F, Zhao TJ, Fang XH, Li YQ, Sui SF. 2008. TINY, a dehydration-responsive element (DRE)-binding protein-like transcription factor connecting the DRE- and ethylene-responsive element-mediated signaling pathways in Arabidopsis. Journal of Biological Chemistry 283, 6261-6271.

61. Thomashow MF. 1999. Plant cold acclimation: Freezing tolerance genes and regulatory mechanisms. Annual Review of Plant Physiology and Plant Molecular Biology 50, 571-599.

62. Tsutsui T, Kato W, Asada Y, Sako K, Sato T, Sonoda Y, Kidokoro S, Yamaguchi-Shinozaki K, Tamaoki M, Arakawa K, Ichikawa T, Nakazawa M, Seki M, Shinozaki K, Matsui M, Ikeda A, Yamaguchi J. 2009. DEAR1, a transcriptional repressor of DREB protein that mediates plant defense and freezing stress responses in Arabidopsis. Journal of Plant Research 122, 633-643.

63. Van der Does D, Leon-Reyes A, Koornneef A, Van Verk MC, Rodenburg N, Pauwels L, Goossens A, Körbes AP, Memelink J, Ritsema T, Van Wees SCM, Pieterse CMJ. 2013. Salicylic Acid Suppresses Jasmonic Acid Signaling Downstream of SCF^{COI1}-JAZ by Targeting GCC Promoter Motifs via Transcription Factor ORA59. Plant Cell 25, 744-761.

64. Wan LY, Zhang JF, Zhang HW, Zhang ZJ, Quan RD, Zhou SR, Huang RF. 2011a. Transcriptional Activation of OsDERF1 in OsERF3 and OsAP2-39 Negatively Modulates Ethylene Synthesis and Drought Tolerance in Rice. Plos One 6, 14.

65. Wang QY, Guan YC, Wu YR, Chen HL, Chen F, Chu CC. 2008. Overexpression of a rice OsDREB1F gene increases salt, drought, and low temperature tolerance in both Arabidopsis and rice. Plant Molecular Biology 67, 589-602.

66. Wang X, Hou C, Zheng K, Li Q, Chen S, Wang S. 2017. Overexpression of ERF96, a small ethylene response factor gene, enhances salt tolerance in Arabidopsis. Biologia Plantarum 61, 693-701.

67. Wawrzynska A, Christiansen KM, Lan Y, Rodibaugh NL, Innes RW. 2008. Powdery Mildew Resistance Conferred by Loss of the EDR1 Protein Kinase is Supressed by a Missense Mutation in KEG, a Regulator of ABA Signaling. Plant Physiology 148, 1510-1522

68. Xiao GQ, Qin H, Zhou JH, Quan RD, Lu XY, Huang RF, Zhang HW. 2016. OsERF2 controls rice root growth and hormone responses through tuning expression of key genes involved in hormone signaling and sucrose metabolism. Plant Molecular Biology 90, 293-302.

69. Yang HJ, Shen H, Chen L, Xing YY, Wang ZY, Zhang JL, Hong MM. 2002. The OsEBP-89 gene of rice encodes a putative EREBP transcription factor and is temporally expressed in developing endosperm and intercalary meristem. Plant Molecular Biology 50, 379-391.

70. Yang J, Ordiz MI, Jaworski JG, Beachy RN. 2011. Induced accumulation of cuticular waxes enhances drought tolerance in Arabidopsis by changes in development of stomata. Plant Physiology and Biochemistry 49, 1448-1455.

71. Yang Z, Tian L, Latoszek-Green M, Brown D, Wu K 2005. Arabidopsis ERF4 is a transcriptional repressor capable of modulating ethylene and abscisic acid responses. Plant Molecular Biology 58, 585-596.

72. Yao Y, He RJ, Xie QL, Zhao XH, Deng XM, He JB, Song L, He J, Marchant A, Chen XY, Wu AM. 2017. ETHYLENE RESPONSE FACTOR 74 (ERF74) plays an essential role in controlling a respiratory burst oxidase homolog D (RbohD)-dependent mechanism in response to different stresses in Arabidopsis. New Phytologist 213, 1667-1681.

73. Yu YW, Yang DX, Zhou SR, Gu JT, Wang FR, Dong JG, Huang RF. 2017. The ethylene response factor OsERF109 negatively affects ethylene biosynthesis and drought tolerance in rice. Protoplasma 254, 401-408.

74. Zhang H, Hong Y, Huang L, Li D, Song F. 2016. Arabidopsis AtERF014 acts as a dual regulator that differentially modulates immunity against *Pseudomonas syringae* pv. *tomato* and *Botrytis cinerea*. Scientific Reports 6, 30251.

75. Zhang HJ, Huang L, Dai Y, Liu SX, Hong YB, Tian LM, Huang LH, Cao ZY, Li DY, Song FM. 2015. Arabidopsis AtERF15 positively regulates immunity against *Pseudomonas syringae* pv. *tomato* DC3000 and *Botrytis cinerea*. Frontiers in Plant Science 6, 13.

76. Zhang Z, Wang J, Zhang R, Huang R. 2012. The ethylene response factor AtERF98 enhances tolerance to salt through the transcriptional activation of ascorbic acid synthesis in Arabidopsis. Plant Journal 71, 273-282.

77. Zhang XX, Tang YJ, Ma QB, Yang CY, Mu YH, Suo HC, Luo LH, Nian H. 2013. OsDREB2A, a Rice Transcription Factor, Significantly Affects Salt Tolerance in Transgenic Soybean. Plos One 8.

78. Zhao JL, Zhang SH, Yang TF, Zeng ZC, Huang ZH, Liu Q, Wang XF, Leach J, Leung H, Liu B. 2015. Global transcriptional profiling of a cold-tolerant rice variety under moderate cold stress reveals different cold stress response mechanisms. Physiologia Plantarum 154, 381-394.

79. Zhu L, Liu D, Li Y, Li N. 2012. Functional phosphoproteomic analysis reveals that a Ser62-phosphorylated isoform of Ethylene Response Factor 110 is involved in Arabidopsis bolting. Plant Physiology 161, 904-917.

80. Zhu Q, Zhang J, Gao X, Tong J, Xiao L, Li W, Zhang H. 2010. The Arabidopsis AP2/ERF transcription factor RAP2.6 participates in ABA, salt and osmotic stress responses. Gene 457, 1-12.

81. Zhuang J, Jiang HH, Wang F, Peng RH, Yao QH, Xiong AS. 2013. A Rice OsAP23, Functioning as an AP2/ERF Transcription Factor, Reduces Salt Tolerance in Transgenic Arabidopsis. Plant Molecular Biology Reporter 31, 1336-1345.

82. Zou Y, Chintamanani S, He P, Fukushige , Yu L, Shao M, Zhu L, Hildebrand DF, Tang X, Zhou J-M. 2015. A gain-of-function mutation in *Msl10* triggers cell death and wound-induced hyperaccumulation of jasmonic acid in Arabidopsis. Journal of Integrative Plant Biology 58, 600-609.
