# Supplementary material for: Computational Disorder Analysis in Ethylene Response Factors Uncovers Binding Motifs Critical to Their Diverse Functions
Source: Int J Mol Sci. 2019 Dec 20;21(1):74. doi: 10.3390/ijms21010074 (PMC6981732; doi:10.3390/ijms21010074)
Supplement: Supplementary file 1 [file ijms-21-00074-s001.zip › ijms-650542-supplementary-XML/Supplementary Table S1.pdf]

Supplemental Table S1

Group I	Group by Nakano and (Sakuma)†	Other names	Stress/hormone function‡
AT1G22190.1	Ib (A-6)	AtERF058	
AT1G36060.1	Ib (A-6)	AtERF055	
AT1G64380.1	Ib (A-6)	AtERF061	
AT1G78080.1	Ib (A-6)	RAP2.4, WIND1, AtERF059	wounding, cytokinin[26]
AT2G22200.1	Ib (A-6)	AtERF056	
AT4G39780.1	Ib (A-6)	AtERF060	
AT5G65130.1	Ib (A-6)	AtERF057	
Os01g66270.1	IIc	OsERF017	
Os02g51670.1	Ib	OsERF051	
Os03g09170.1	Ib	OsERF047	
Os04g44670.1①	Ia	OsERF045	
Os05g49700.1	Ib	OsERF052	
Os05g34730.1	IIc	OsERF131, OsSERF1	salt[54]
Os06g11860.1	Ib	OsERF120	
Os08g31580.1	Ib	OsERF048, OsDRAP1	drought[29]
Os09g20350.1	Ib	OsERF050	
Os10g22600.1	Ib	OsERF051	
Pp1s21_214V6.1			
Pp1s79_25V6.1			
Pp1s187_47V6.1			
Pp1s335_8V6.1			

Group IIa	Group by Nakano and (Sakuma)	Other names	Stress/hormone function‡
AT1G01250.1	IIIa (A-4)	AtERF023	
AT1G19210.1	IIb	AtERF017	
AT1G22810.1	IIc	AtERF019	drought[52]
AT1G33760.1	IIIa (A-4)	AtERF022	
AT1G46768.1	IIa	RAP2.1, AtERF006	cold, drought[14]
AT1G71520.1	IIc	AtERF020	
AT1G74930.1	IIb	AtERF018	
AT2G23340.1	IIa	AtERF008	
AT3G50260.1	IIa	AtERF011, DEAR1, CEJ1	Biotic, cold, JA[44], SA[62]
AT4G06746.1	IIa	RAP2.9, AtERF007	
AT4G36900.1	IIa	RAP2.10, AtERF009	
AT5G21960.1	IIb	AtERF016	
AT5G67190.1	IIa	AtERF010	
Os02g54050.1	IIb	OsERF011	
Os04g55520.1	IIa	OsERF008	
Os06g07030.1	IIa	OsERF007	
Os06g09717.1			
Os06g09760.1	IIb	OsERF016	
Os06g09790.1	IIb	OsERF015	
Os06g09810.1	IIb	OsERF014	
Os06g11940.1	IIb	OsERF013	
Pp1s8_127V6.1			
Pp1s15_186V6.1			
Pp1s15_189V6.1			
Pp1s21_320V6.1			
Pp1s29_45V6.1			
Pp1s55_253V6.1			
Pp1s55_256V6.1			
Pp1s60_269V6.1			
Pp1s117_23V6.1			
Pp1s133_11V6.1			
Pp1s155_21V6.1			
Pp1s183_112V6.1			
Pp1s215_64V6.1			
Pp1s259_94V6.1			
Pp1s259_96V6.1			
Pp1s259_97V6.1			
Pp1s263_66V6.1			
Pp1s263_67V6.1			
Pp1s276_3V6.1			
Pp1s480_12V6.1			
Pp1s480_16V6.1			

Group IIb	Group by Nakano and (Sakuma)	Other names	Stress/hormone function‡
AT1G44830.1	IIb (A-5)	AtERF014	biotic[74], JA, SA[5]
AT1G21910.1	IIb (A-5)	AtERF016	
AT1G77640.1	IIb (A-5)	AtERF013	
AT4G31060.1	IIb (A-5)	AtERF015	
Os03g15660.1	IIb	OsERF009	
Os06g10780.1	IIb	OsERF119	
Pp1s11_280v6.1			
Pp1s87_98v6.1			
Pp1s119_43v6.1			
Pp1s86_33v6.1			
Pp1s373_18v6.1			

Group IIIa	Group by Nakano and (Sakuma)	Other names	Stress/hormone function‡
AT1G71450.1	IIIa (A-4)	FUF1, AtERF021	Senescence, Abscission[8]
AT5G07580.1	IXb (B-3b)	AtERF106	
AT5G61590.1	IXb (B-3b)	AtERF107	
Os02g35240.1	IIIb	OsERF021	
Os11g13840.1	IIIb	OsERF019	
Pp1s115_154V6.1			
Pp1s357_12V6.1			

Group IIIb	Group by Nakano and (Sakuma)	Other names	Stress/hormone function‡
AT1G12630.1	IIIb (A-4)	AtERF027	
AT1G63040.1	IIIb (A-4)	AtERF026	biotic[23]
AT2G36450.1	IIIb (A-4)	HARDY, AtERF024	water, salt, biotic[23], drought[1]
AT5G52020.1	IIIb (A-4)	AtERF025	biotic[23]
Os02g45420.1	IIIb	OsERF020	
Os10g38000.1	IIIc	OsERF018	

Group IIIc	Group by Nakano and (Sakuma)	Other names	Stress/hormone function
AT1G12610.1	IIIc (A-1)	AtERF033	
AT1G63030.1	IIIc (A-1)	DDF1, AtERF032	GA, salt, cold, drought[30]
AT4G25470.1	IIIc (A-1)	CBF2, DREB1C, AtERF030	cold[36]
AT4G25480.1	IIIc (A-1)	CBF3, DREB1A, AtERF031	cold[36]
AT4G25490.1	IIIc (A-1)	CBF1, DREB1B, AtERF029	cold[61], drought
AT5G51990.1	IIIc (A-1)	CBF4, DREB1D, AtERF028	drought[20]
Os01g73770.1	IIIc	OsERF027, OsDREB1F	drought, salt, cold[65]
Os02g45450.1	IIIc	OsERF025	
Os04g48350.1	IIIc	OsERF030	
Os06g03670.1	IIIc	OsDREB1C, OsERF026	cold, drought[15]
Os08g43200.1	IIIc	OsERF028	
Os08g43210.1	IIIc	OsERF029	
Os09g35010.1	IIIc	OsDREB1B, OsERF031	cold[7], drought[17], salt[25]
Os09g35020.1	IIIc	OsERF133	
Os09g35030.1	IIIc	OsDREB1A, OsERF024	cold, drought[7], salt[25]

Group IIId	Group by Nakano and (Sakuma)	Other names	Stress/hormone function
AT1G71130.1	VI (B-5)	CRF8, AtERF070	Cytokinin[13], phosphate, GA[50]
AT1G77200.1	IIIe (A-4)	AtERF037	
AT2G25820.1	IIIe (A-4)	AtERF042	
AT2G44940.1	IIId (A-1)	AtERF034	
AT3G16280.1	IIIe (A-4)	AtERF036	
AT3G60490.1	IIId (A-4)	AtERF035	
AT4G32800.1	IIIe (A-4)	AtERF043	
AT5G11590.1	IIIe (A-4)	AtERF041	
AT5G25810.1	IIIe (A-4)	TINY, AtERF040	ABA, ethylene, MeJA, cold, drought[60]
Os01g10370.1	IIId	OsERF039	
Os02g13710.1	IIIe	OsERF038	
Os02g43940.1	IIId	OsERF032	
Os02g43970.1	IIId	OsERF035	
Os03g07830.1	IVb	OsERF041	
Os04g46400.1	IIId	OsERF033	
Os04g46410.1	IIId	OsERF037	
Os04g46440.1	IIId	OsERF034	
Os05g28350.1	IVb	OsERF117	
Os06g36000.1	IIIe	OsERF122	
Os10g41130.1	IIId	OsERF036	
Pp1s14_196V6.1①			
Pp1s28_144V6.1			

Group IV	Group by Nakano and (Sakuma)	Other names	Stress/hormone function‡
AT1G75490.1	IVb (A-2)	DREB2D, AtERF049	heat, drought, salt[51]
AT2G38340.1	IVa (A-2)	DREB2E, AtERF046	
AT2G40220.1	IVb (A-3)	ABI4, AtERF052	salt, ABA, GA[57], water, osmotic
AT2G40340.1	IVa (A-2)	DREB2C, AtERF048	heat[39], ABA[27], salt[58], oxidative[24]
AT2G40350.1	IVa (A-2)	DREB2H, AtERF047	
AT3G11020.1	IVa (A-2)	DREB2B, AtERF044	salt, water[45]
AT3G23220.1	IXc (B-3c)	AtERF095	
AT3G57600.1	IVb (A-2)	DREB2F, AtERF051	
AT5G05410.1	IVa (A-2)	DREB2A, AtERF045	salt, water[45]
AT5G18450.1	IVb (A-2)	DREB2G, AtERF050	
Os01g07120.1	IVa	OsDREB2A, OsERF040	cold[78], salt[77], ABA, drought[12]
Os03g64260.1	IXc	OsERF083	
Os05g27930.1	IVb	OsERF042	
Os05g39590.1	IVb	OsERF043	
Os08g35240.1①	IIb	OsERF012, OsDERF1	drought, ethylene[64]
Os08g45110.1	IVb	OsERF044	
Pp1s23_27V6.1			
Pp1s81_44V6.1			
Pp1s162_92V6.1			
Pp1s292_5V6.1			

Group V	Group by Nakano and (Sakuma)	Other names	Stress/hormone function‡
AT1G15360.1	Va (B-6)	WIN1, SHN1, AtERF001	biotic, water, drought[70]
AT4G13620.1	Ib (A-6)	AtERF062	
AT4G17490.1	IXb (B-3b)	AtERF6, ATERF103	cold[4]
AT5G11190.1	Va (B-6)	SHN2, AtERF004	
AT5G25190.1	Va (B-6)	AtERF003	
AT5G25390.1	Va (B-6)	SHN3, AtERF005	
AT5G47230.1	IXb (B-3b)	AtERF5, AtERF102	cold[4]
Os02g10760.1	Va	OsERF003	
Os02g55380.1	Va	OsERF127	
Os04g56150.1	Va	OsERF129	
Os06g08340.1	Va	OsERF002	ABA, ethylene[68]
Os06g40150.1	Va	OsERF001	
Os08g42550.1	Xc	OsERF106	
Pp1s17_332V6.1			
Pp1s107_45V6.1			
Pp1s174_79V6.1			
Pp1s179_56V6.1			
Pp1s225_102V6.1			
Pp1s296_69V6.1			

Group VI	Group by Nakano and (Sakuma)	Other names	Stress/hormone function‡
AT2G33710.1①	Xc (B-4)	AtERF112	
AT5G50080.1	Xc (B-4)	AtERF110	Development[79]
Os01g12440.1	VI	OsERF053	
Os01g46870.1	VI	OsERF054	
Os03g60120.1	VI	OsERF058	
Os05g25260.1	VI	OsERF056	
Os06g06540.1	VI	OsERF055	
Os07g12510.1	VI	OsERF057	

Group VI-L	Group by Nakano and (Sakuma)	Other names	Stress/hormone function‡
AT1G22985.1	VI (B-5)	CRF7, AtERF069	cytokinin[13]
AT1G25470.1	VI-L (B-6)	AtERF116	
AT1G49120.1	VI-L (B-6)	AtERF117	
AT1G68550.1	VI-L (B-6)	AtERF118	
AT2G46310.1	VI (B-5)	CRF5, AtERF068	cytokinin[49]
AT3G25890.1	VI-L (B-6)	AtERF119	
AT3G61630.1	VI (B-5)	CRF6, AtERF067	Cytokinin[49], oxidative, heat, salt
AT4G11140.1	VI (B-5)	CRF1, AtERF063	cytokinin[49]
AT4G23750.1	VI (B-5)	CRF2, AtERF064	cytokinin[49]
AT4G27950.1	VI (B-5)	CRF4, AtERF066	
AT5G53290.1	VI (B-5)	CRF3,AtERF065	cytokinin[13]
Os01g04020.1	VI-L	OsERF108	
Os08g27220.1	VI-L	OsERF138	ethylene, drought
Os09g13940.1	VI-L	OsERF109	ethylene, drought[73]
Pp1s130_106V6.1			
Pp1s138_190V6.1			

Group VIIa	Group by Nakano and (Sakuma)	Other names	Stress/hormone function‡
AT1G53910.1	VIIa (B-2)	RAP2.12, AtERF074	drought, heat, light[72], oxidative, osmotic, low oxygen[47]
AT1G72360.1	VIIa (B-2)	AtERF073	
AT2G47520.1	VIIa (B-2)	AtERF071	
AT3G14230.1	VIIa (B-2)	RAP2.2, AtERF075	biotic, oxidative, osmotic[47]
AT3G16770.1	VIIa (B-2)	RAP2.3, AtEBP, AtERF072	GA, oxidative, osmotic[47]
Os02g54160.1	VIIa	OsEREBP1, OsERF070	biotic[55]
Os03g08470.1	VIIa	OsERF062	
Os05g29810.1	VIIa	OsERF061	
Os06g09390.1	VIIa	OsERF071	drought[35]
Os07g42510.1	VIIa	OsERF065	
Os09g26420.1	VIIa	OsERF072, OsEBP2, OsBIERF1	biotic[40], ethylene, JA, ABA[53], salt, cold, drought, wounding[6]
Pp1s140_167V6.1①			

Group VIIb	Group by Nakano and (Sakuma)	Other names	Stress/hormone function‡
AT5G07310.1	Xa (B-4)	AtERF115	
AT5G61890.1	Xa (B-4)	AtERF114	biotic[23]
Os02g38090.1	VIIIb	OsERF079	
Os09g11460.1	VIIb	OsERF073	
Os09g11480.1	VIIa	OsERF063	
Pp1s90_184V6.1			
Pp1s105_212V6.1			
Pp1s124_33V6.1			
Pp1s127_75V6.1			
Pp1s149_253V6.1			
Pp1s487_24V6.1			

Group VIIc	Group by Nakano and (Sakuma)	Other names	Stress/hormone function‡
AT4G34410.1	Xb (B-3)	RRTF1, AtERF109	oxidative, IAA/Auxin, JA, biotic[23]
Os02g06330.1	VIIIa	OsERF132	
Os03g08460.1	VIIa	OSEBP89, OsERF060	ethylene/development[69]
Os03g08490.1	VIIa	OsERF069	
Os03g08500.1	VIIa	OsERF064, OsBIERF4	biotic, salt, cold, drought, wounding[6]
Os04g34970.1	Xa	OsERF100	
Os04g52090.1	VIIIa	OsERF077	
Os06g47590.1	VIIIa	OsERF121	
Pp1s1_34V6.1			
Pp1s13_457V6.1			
Pp1s25_13V6.1			
Pp1s26_306V6.1			
Pp1s37_293V6.1			
Pp1s45_159V6.1			
Pp1s61_230V6.1			
Pp1s83_10V6.1			
Pp1s199_50V6.1			
Pp1s296_32V6.1			

Group VIIIa	Group by Nakano and (Sakuma)	Other names	Stress/hormone function‡
AT1G28360.1	VIIIa (B-1)	AtERF12, AtERF081	
AT1G28370.1	VIIIa (B-1)	AtERF11, AtERF076	GA, ethylene biosynthesis[38]
AT1G50640.1	VIIIa (B-1)	AtERF3, AtERF082	salt[59]
AT1G53170.1	VIIIa (B-1)	AtERF8, AtERF079	
AT3G15210.1	VIIIa (B-1)	RAP2.5, AtERF4, AtERF078	biotic[42], JA, ethylene, ABA[71], oxidative, salt[59]
AT3G20310.1	VIIIa (B-1)	AtERF7, AtERF083	
AT5G44210.1	VIIIa (B-1)	AtERF9, AtERF080	biotic , JA[44]
Os01g58420.1	VIIIa	OsERF3, OsERF075, OsBIERF2	biotic[4], drought[28]
Os05g41760.1	VIIIa	OsERF130	
Os05g41780.1	VIIIa	OsERF074	
Pp1s7_472V6.1			
Pp1s7_481V6.1			
Pp1s21_239V6.1			
Pp1s21_247V6.1			
Pp1s23_8V6.1			
Pp1s30_284V6.1			
Pp1s38_262V6.1			
Pp1s126_30V6.1			
Pp1s293_53V6.1			
Pp1s293_60V6.1			
Pp1s340_16V6.1			

Group VIIIb	Group by Nakano and (Sakuma)	Other names	Stress/hormone function‡
AT1G12890.1	VIIIb (B-1)	AtERF088	
AT1G28160.1	VIIIb (B-1)	AtERF087	
AT1G80580.1	VIIIb (B-5)	AtERF084	
AT5G13910.1	VIIIb	LEP, AtERF085	Biotic, GA[7]
AT5G18560.1	VIIIb (B-1)	AtERF086	
Os02g32040.1	VIIIb	OsERF081	
Os04g32790.1	VIIIb	OsERF082	
Os07g47330.1	VIIIb	FZP, OsERF078	plant development[33]
Os08g07700.1	VIIIb	OsERF080	
Os12g41030.1	XI	OsERF110	
Os12g41060.1	XI	OsERF112	
Pp1s5_432V6.1			
Pp1s6_75V6.1			
Pp1s74_86V6.1			
Pp1s221_67V6.1①			

Group VIIIc	Group by Nakano and (Sakuma)	Other names	Stress/hormone function‡
AT1G12980.1	VIIIb (B-1)	ESR1, DRN, AtERF089	auxin[11]
AT1G24590.1	VIIIb (B-1)	AtERF090, ESR2, DRNL	cytokinin[16]
Os06g06970.1			
Pp1s15_480V6.1			
Pp1s145_10V6.1			
Pp1s204_42V6.1			
Pp1s233_111V6.1			
Pp1s276_72V6.1			

Group IXa	Group by Nakano and (Sakuma)	Other names	Stress/hormone function‡
AT1G03800.1	VIIIa (B-1)	AtERF10, AtERF077	Aux/IAA[9]
AT4G17500.1	IXa (B-3a)	AtERF1, AtERF100	ABA[67], JA[48]
AT5G47220.1	IXa (B-3a)	AtERF2	
AT5G51190.1	IXb (B-3b)	AtERF105	cold[4]
AT5G61600.1	IXb (B-3b)	AtERF104	cold[4]
Os01g54890.1	IXa	OsERF092	biotic, ABA[41]
Os07g47790.1	VIIa	OsERF067	
Os10g25170.1	VIIa	OsERF059	
Pp1s14_458V6.1			
Pp1s15_234V6.1			
Pp1s41_212V6.1			
Pp1s112_23V6.1			
Pp1s132_34V6.1			
Pp1s181_69V6.1			
Pp1s276_50V6.1			

Group IXb	Group by Nakano and (Sakuma)	Other names	Stress/hormone function‡
AT5G64750.1	Xa (B-4)	ABR1, AtERF111	ABA, biotic[23], osmotic[46], salt[10]
Os01g21120.1	VIIa	OsERF068	
Os02g09650.1	VIIIa	OsERF134	
Os02g43820.1	IXb	OsERF095	
Os04g46240.1	IXb	OsERF097	
Os04g46250.1	IXb	OsERF094	
Pp1s58_260V6.1			
Pp1s79_169V6.1			
Pp1s243_70V6.1			

Group IXc	Group by Nakano and (Sakuma)	Other names	Stress/hormone function‡
AT1G06160.1	IXc (B-3c)	ORA59, AtERF094	JA[48], SA[63]
AT2G31230.1	IXc (B-3c)	AtERF15, AtERF093	biotic[75], ABA[36]
AT3G23230.1	IXc (B-3c)	TDR1, AtERF098	biotic, salt, oxidative[76]
AT3G23240.1	IXc (B-3c)	ERF1, AtERF092	biotic[3], ethylene, auxin[43]
AT4G18450.1	IXc (B-3c)	AtERF091	
Os05g37640.1	IXc	OsERF085	
Os07g22730.1	IXc	OsERF136	
Os07g22770.1	IXc	OsERF086, OsDERF2	drought[19]
Os09g39810.1	IXc	OsERF123	
Os09g39850.1	IXc	OsERF087	
Os10g30840.1	IXc	OsERF089	
Pp1s2_404V6.1			
Pp1s19_272V6.1			
Pp1s20_184V6.1			
Pp1s38_257V6.1			
Pp1s121_50V6.1			
Pp1s265_50V6.1			
Pp1s345_35V6.1			

Group IXd	Group by Nakano and (Sakuma)	Other names	Stress/hormone function‡
AT1G04370.1	IXc (B-3c)	AtERF14	
AT2G20880.1	Ia (A-6)	AtERF053	heat, ABA[21]
AT4G28140.1	Ia (A-6)	AtERF054	
AT5G43410.1	IXc (B-3c)	AtERF096	ABA, JA, SA, biotic, salt[66]
Os02g42585.1			
Os03g05590.1	IXc	OsERF088, OsAP23	salt[81]
Os04g18650.1	IXc	OsERF128	
Os05g49010.1	IXc	OsERF084	
Os08g44960.1	IXc	OsERF090	
Pp1s53_80V6.1			
Pp1s60_148V6.1			
Pp1s72_230V6.1			
Pp1s120_13V6.1			
Pp1s240_84V6.1①			

Group IXe	Group by Nakano and (Sakuma)	Other names	Stress/hormone function‡
AT2G44840.1	IXa (B-3a)	AtERF13, AtERF099	ABA[37]
Os01g59780.1			
Os03g07940.1			
Os06g44750.1			
Os07g03250.1			
Os08g34360.1			
Os10g41330.1	IXb	OsERF096	
Pp1s2_410V6.1			

Group Xa	Group by Nakano and (Sakuma)	Other names	Stress/hormone function‡
AT1G43160.1	Xa (B-4)	RAP2.6, AtERF108	biotic[2], ABA[18], JA, SA, cold, osmotic[80] salt, water, wounding[82]
AT5G13330.1	Xa (B-4)	AtERF113	
Os01g64790.1	Xa	OsERF099, OsEREBP2	salt, cold, ABA, drought[56]
Os02g34260.1	Xa	OsERF098	
Os02g34270.1	Xa	OsERF125	
Os02g43790.1	IXa	OsERF091, OsBIERF3	biotic, salt, cold, drought, wounding[6]
Os03g22170.1	VIIa	OsERF066	
Os04g46220.1	IXa	OsERF1	biotic, ethylene/development[22]
Os05g36100.1	Xc	OsERF105	
Pp1s31_112V6.1			
Pp1s67_144V6.1①			
Pp1s117_32V6.1			
Pp1s136_219V6.1			
Pp1s160_12V6.1			
Pp1s259_104V6.1			

Group Xb	Group by Nakano and (Sakuma)	Other names	Stress/hormone function‡
AT2G20350.1	Xb-L (B-6)	AtERF120	
AT5G19790.1	Vb (B-6)	RAP2.11, AtERF002	abiotic stress[32]
AT5G67000.1	Xb-L (B-6)	AtERF122	
AT5G67010.1	Xb-L (B-6)	AtERF121	
Os02g52670.1	Xb	OsERF103	
Os04g57340.1	VIIIa	OsERF076	
Os07g10410.1	Vb	OsERF005	
Os07g38750.1	Vb	OsERF006	
Os08g36920.1	Xb	OsERF104	
Os09g28440.1	Xb	OsERF102	
Os12g39330.1	Vb	OsERF004	
Pp1s64_47V6.1			
Pp1s195_38V6.1			

Group Xc	Group by Nakano and (Sakuma)	Other names	Stress/hormone function‡
AT2G35700.1	IIId (A-4)	AtERF038	development[34]
AT4G16750.1	IIId (A-4)	AtERF039	
Os02g32140.1	Xc	OsERF107	
Os04g32620.1	Xa	OsERF101, OsRap2.6	
Os11g06770.1	Xa	OsERF118	
Pp1s6_391V6.1			
Pp1s289_49V6.1			

Individuals	Group by Nakano and (Sakuma)	Other names	Stress/hormone function‡
Pp1s1_860V6.1	N/A	N/A	N/A
Pp1s107_32V6.1	N/A	N/A	N/A
Pp1s60_228V6.1	N/A	N/A	N/A
Pp1s6_134V6.1	N/A	N/A	N/A

New and previous grouping of ERFs (where applicable) are given along with alternative names for these genes. The stress and/or hormonal pathways that representative genes in the group (noted by an asterisk) have been linked with are given and represented pictorially in Figure 1. More distantly related genes that have uncertain group membership status are labelled ①. On the phylogenetic trees they occupy a position that is basal to the rest of the members of the group. They have also been tested for membership of other groups but appear not to fit with any other group.
These genes have therefore tentatively been placed in these groups but reflect the extremely diverse nature of the ERF protein family.

† Grouping of this ERF by Nakano et al. (2006) where it exists while its grouping by Sakuma et al. (2002) where it exists is given in brackets.

‡ Functions associated with a particular hormone and/or the type of stress. Superscripted numbers refer to source references for the information linking the gene to the stipulated stress, hormone or function. These are provided in Supplemental File S1.
