# Supplementary material for: Computational Disorder Analysis in Ethylene Response Factors Uncovers Binding Motifs Critical to Their Diverse Functions
Source: Int J Mol Sci. 2019 Dec 20;21(1):74. doi: 10.3390/ijms21010074 (PMC6981732; doi:10.3390/ijms21010074)
Supplement: Supplementary file 1 [file ijms-21-00074-s001.zip › ijms-650542-supplementary-XML/Supplementary Table S2.pdf]

Supplementary Table S2. Summary of conserved MoRFs in carboxy and amino terminal regions outside of the AP2 domains

Group I

I N1*

AT1G64380.1	DIIEKFhL
AT1G36060.1	ELMEALqP
AT1G22190.1	ELMEALlP
AT5G65130.1	EFMEALeP
Os06g11860.1	ELMKALeP
AT4G39780.1	ELMKALqP
AT1G78080.1	ELMDALvP
Os09g20350.1	ELMRALeP
Os03g09170.1	ELMGALaP
Os02g51670.1	ELMKALeP
Consensus sequences	[ED]-[LIF]-[MI]-[EDKRG]-[AK]-[LF]-x-[PL]

I C1

AT1G64380.1	FlLarmPSfdpE.LIWE
AT2G22200.1	FgLrkfPSv..E.IDWD
AT1G36060.1	FmLgkcPSy..E.IDWA
AT1G22190.1	NaLekyPSy..E.IDWD
Pp1s187_47V6.1	LlLgsvPSse.EqVDWS
AT5G65130.1	MgLhkyPSl..E.IDWD
AT4G39780.1	FgLekfPSv..E.IDWD
Os06g11860.1	FhLykcPSw..E.IDWD
AT1G78080.1	FsLekyPSy..E.IDWD
Os10g22600.1	FaLrryPSw..E.IDWD
Os09g20350.1	FaLtkyPSy..E.IDWD
Os03g09170.1	FlLhkyPSl..E.IDWD
Os08g31580.1	FaLtkyPSy..E.IDWD
Os02g51670.1	FhLrkyPSw..E.IDWD
Consensus sequences	L-x(3)-P-[SN]-x(1,3)-E-x(0,1)-[VLI]-[DI]-W

Subgroup IIa

IIa C1*

AT4G06746.1	IRkKAaEVGarVDA
AT4G36900.1	IRrKAaEVGaqVDA
Os04g55520.1	IRkKAiEVGsrVDA
AT3G50260.1	IRkKAtEVGaqVDA
AT2G23340.1	IRrKAaEVGaqVDA
AT5G67190.1	IRkKAtEVGaqVDA
Os06g07030.1	IRkKAaEVGarVDA
AT1G46768.1	IReKAaEVGarVDA
Consensus sequences	I-R-x-K-A-x-E-V-G-x(2)-V-D-A

IIa C2*

AT4G06746.1	LDMNklP...Eay..G
AT4G36900.1	VDLNklPpd.EtsddD
Os04g55520.1	PDLNqaPps.EnsdaE
AT3G50260.1	VDLNklPpd.EnlddD
AT2G23340.1	VDLNklPpd.EnsdgD
AT5G67190.1	VDLNklPpdPEssdeE
Os06g07030.1	PDLNrePpt.DtsddE
AT1G46768.1	PDLNqiP...Esg..D
Consensus sequences	D-[ML]-N-x(2)-P-x(0,3)-[DE] EAR Motifs

IIa C3

AT1G71520.1	MEEedkLsI
Pp1s480_16V6.1	LEEve.LaW
AT1G74930.1	LDEsl.IlW
Pp1s60_269V6.1	LEDvd.LaF
Pp1s8_127V6.1	LEDvg.LaW
Pp1s55_256V6.1	LEDve.LaW
Pp1s155_21V6.1	LDDve.LaW
Consensus sequences	[LM]-[ED]-[ED]-x(2,3)-[IL]-x-[FWI]

IIa C4

Pp1s259_96V6.1	EDLlqeL
Pp1s480_16V6.1	DDInl.I
Pp1s183_112V6.1	DDFne.W
Pp1s155_21V6.1	DDLnf.L
AT1G01250.1	DDFwegI
Pp1s8_127V6.1	DDIkf.L
Os06g11940.1	DDLav.W
Pp1s259_97V6.1	EDLlqeL
Pp1s55_256V6.1	DDInf.L
Consensus sequences	[ED]-D-[ILF]-x(2,3)-[LIW]

IIa C5

Pp1s276_3V6.1	LWcF
Pp1s15_189V6.1	LWcF
AT1G19210.1	LWnF
AT1G22810.1	VYdY
Os02g54050.1	LWsF
AT1G01250.1	LYqY
Pp1s259_94V6.1	LWcF
Pp1s15_186V6.1	LWcF
AT5G21960.1	LWnF
Pp1s259_97V6.1	LWsF
AT1G71520.1	VYdY
Pp1s259_96V6.1	LWsF
AT1G74930.1	LWdF
Pp1s117_23V6.1	LWsF
Os06g09810.1	VYaF
Pp1s55_253V6.1	LWfF
Pp1s133_11V6.1	LWsY
Os06g11940.1	VYaF
Os06g09717.1	LWsF
Consensus sequences	[LV]-[WYF]-x-[FY] LWSY Motif

Subgroup IIb

IIb C1*

Pp1s119_43V6.1	PREIQFweVLnR
Pp1s11_280V6.1	PREIQFavVLdR
Pp1s87_98V6.1	PKEIQFavVLnR
Pp1s86_33V6.1	PREIQFleVLnR
Consensus sequences	P-[RK]-EIQF-x(2)-VL-x-R

IIb C2*

AT1G44830.1	EEgDIr.LWNF
Os06g10780.1	EEgDIa.LWSF
AT1G21910.1	EDaDIp.LWSF
AT1G77640.1	DDvDIp.LWSF
Os03g15660.1	EDgELmrLWSF
Consensus sequences	[ED]-[ED]-x-[ED]-[LI]-x(1,2)-L-W-[SN]-F LWSY motif

Subgroup IIIa

IIIa C1

AT1G71450.1	LWD
Os11g13840.1	LWD
Os02g35240.1	LWD
Consensus sequences	L-W-D

Subgroup IIIb

IIIb C1

Os02g45420.1	EsLWsY
AT1G12630.1	EdLWgY
AT2G36450.1	DyLWnF
AT5G52020.1	DnLWsY
AT1G63040.1	EsLWsY
Os05g49700.1	ElIWeM
Consensus sequences	[ED]-x-[LI]-W-x[YFM] LWSY-like Motif

Subgroup IIIc

IIIc N1*

Os08g43200.1	KRpAGRtKFqETRHPV
Os09g35010.1	KRpAGRtKFrETRHPV
Os04g48350.1	KRrAGRtKFkETRHPV
AT4G25490.1	KKpAGRkKFrETRHPI
Os09g35020.1	KRpAGRtKFrETRHPV
AT1G63030.1	KKrAGRriFkETRHPI
AT5G51990.1	KKrAGRkKFrETRHPI
Os06g03670.1	KRpAGRtKFrETRHPV
Os01g73770.1	KRrAGRkKFrETRHPV
Os09g35030.1	KRpAGRtKFrETRHPV
AT1G12610.1	KKrAGRvFkETRHPV
AT4G25480.1	KKpAGRkKFrETRHPI
Os02g45450.1	KRrAGRtKFkETRHPV
AT4G25470.1	KKpAGRkKFrETRHPI
Os08g43210.1	KRpAGRtKFhETRHPV
Consensus sequences	K-[RK]-x-A-G-R-x(2)-F-x-E-T-R-H-P-[VI] Positively charged motif prior to AP2 domain

IIIc C1

Os09g35010.1	DMpLWS
Os04g48350.1	EVnLWS
AT1G63030.1	DLsLWS
AT5G51990.1	DVsLWS
AT1G12610.1	DMsLWS
AT4G25470.1	DVsLWS
Os08g43200.1	EMeLWS
AT4G25490.1	DVsLWS
Os09g35020.1	DIaLWS
Os06g03670.1	DVtLWS
Os09g35030.1	DVpLWS
AT4G25480.1	DVsLWS
Os02g45450.1	EVqLWS
Os08g43210.1	DVsLWS
Consensus sequences	[ED]-[VMLI]-x-L-W-S

Subgroup IIId

IIId C1*

AT5G25810.1	EeLgE.IVELPsL
AT4G32800.1	DdLdE.IVKLPsL
Os02g13710.1	DeL.EaIVELPrL
AT1G77200.1	EeLsE.IVELPsL
AT5G11590.1	EeLgE.IVELPsL
AT2G25820.1	E.LgD.IVELPsL
Os06g36000.1	EeLeE.IVELPpI
AT3G16280.1	ErL.EkIVELPnI
Consensus sequences	[ED]-x-L-x-[ED]-x-I-V-[EK]-L-P-x-L EDLL-like motif

IIId C2

Os04g46410.1	DdadDFFFgE
Os04g46440.1	Dlp.DLLL.D
AT4G32800.1	Esn.EFVIfD
Os02g43970.1	DaadDALF.D
Os10g41130.1	Dlp.DLLF.D
Os04g46400.1	Dlp.DLLL.D
AT3G60490.1	Dlp.DLFT.D
AT2G44940.1	Dlp.DLFTdE
AT1G77200.1	EslsEFVYvD
Os02g43940.1	Dlp.DLLL.D
Consensus sequences	[ED]-x(2,3)-[ED]-[LFA]-[LFIV]-x(0,1)-[ED]

IIId C3

AT5G25810.1	DsadLW
Os01g10370.1	ElapLW
Os04g46410.1	EplvLW
Os02g43970.1	EeplLW
Os04g46400.1	Epl.LW
Os10g41130.1	EeplLW
Os04g46440.1	EepmLW
Pp1s14_196V6.1	EfdnLW
AT2G25820.1	Esf.LW
Consensus sequences	[ED]-x(2,3)-L-W

Group IV

IV N1*

Os05g27930.1	Et.IkR.WaE
Os01g07120.1	Et.I.KwWkE
AT3G11020.1	Dr.LkK.WkE
AT2G38340.1	EatLqR.WeE
AT2G40340.1	Ei.L.RqWrE
AT5G05410.1	Er.LkR.WkE
AT2G40350.1	Ei.LrK.WrE
Consensus sequences	[ED]-x(1,2)-[LI]-x(0,1)-[RK]-x(0,1)-W-x-E

IV N2*

Os05g27930.1	KKGcmKGKGGPEN
Pp1s162_92V6.1	KKGamKGKGGPEN
AT1G75490.1	RKGcmRGKGGPDN
Pp1s81_44V6.1	KKGcmKGKGGPEN
AT3G11020.1	KKGcmKGKGGPDN
AT2G40340.1	RKGcmKGKGGPEN
AT2G40350.1	RKGcmKGKGGPEN
AT5G05410.1	KKGcmKGKGGPEN
Pp1s23_27V6.1	KRGcmKGKGGPEN
Os08g45110.1	RKGcmKGKGGPEN
Os01g07120.1	KKGcmAGKGGPEN
AT2G38340.1	KKGcmRGKGGPEN
AT5G18450.1	RKGcmKGKGGPEN
Pp1s292_5V6.1	KKGamKGKGGPEN
Consensus sequences	[KR]-[RK]-G-x(2)-[RKA]-G-K-G-G-P-[ED]-N Positively charged motifs

Group V

V N1*

Pp1s17_332V6.1	MNLTISSIIEQGKPKtKk.WDpCGG..KvKEQaLqL
Pp1s225_102V6.1	MNLTISSILEHGKPKsKtwWDsCGGsnKgREQgLlL
Pp1s174_79V6.1	MNLTISSILEQGRPKsKnwWDpCGGg.KaREQgLqL
Pp1s179_56V6.1	MNLTISSIIEQGKPKaKncWDpCGGg.KiREQgLqL
Consensus sequences	MNLTISSI-[IL]-E[QH]G-[RK]-PK-x-K-x(1,2)-WD-x-CGG-x(0,2)-K-x-[RK]-EQ-x-L-x-L

V C1*

Pp1s17_332V6.1	LQlyPIPPELPWESFAALVKQRATSAAKRARVPSS
Pp1s225_102V6.1	LQlhPIPPELPWESFAALVKQRATSAAKRARVPSS
Pp1s174_79V6.1	LQlyPIPPELPWESFAALVKQRATSAAKRARVPSS
Pp1s179_56V6.1	LQqlPIPPELPWESFAAVVKQRATSAAKRARVPSS
Consensus sequences	LQ-x(2)-PIPPELPWESFAA-[VL]-VKQRATSAAKRARVPSS

V C2

AT5G11190.1	EDqlameMIEEL
Os02g55380.1	EEqhveqMIDEL
Os06g40150.1	EErialqMIEEL
AT1G15360.1	EEkvalqMIEEL
AT5G25390.1	DDqmamqMIEEL
Os06g08340.1	EEqyvdqMIEEL
Os02g10760.1	EEkialqMIEEL
Os04g56150.1	DEyvee.MIqEL
AT5G25190.1	EEdhieqMIEEL
Consensus sequences	[ED]-[ED]-x(4,5)-M-I-x-E-L

Group VI-L

VI-L N1

Os01g04020.1	RrVrVvfeDpdATDsDSDDD
AT1G68550.1	RkVrIivnDpyATDdSSSDE
Os09g13940.1	RkVrVfcsDpdATDsSDEDD
AT1G22985.1	RiVrIsftDmeATDsSSSED
AT3G25890.1	RkLrFvvnDpyATDySSSEE
AT4G27950.1	RlVrVsmtDrdATDsSSDEE
AT1G25470.1	RtVrIvftDpdATDdSSSSS
AT4G23750.1	RiLrIsvtDpdATDsSSDDE
AT1G49120.1	KtIsVicnDpdATDsSSDDE
AT2G46310.1	KiVkIivtDpcATDsSSDEE
AT4G11140.1	RiLrIsvtDpyATDsSSDEE
AT5G53290.1	KkVsIcytDpdATDsSSDED
Os08g27220.1	RrIrIsysDpdATDsDSDSD
AT3G61630.1	RlVrItvtDpfATDsSSDDD
Consensus sequences	[KR]-x-[VLI]-x-[IVF]-x(3)-D-x(2)-A-T-D-x-[SD]-[SD]-[SDE]-[SED]-[SED] Highly charged SDE Motif

VI-L N2

AT3G25890.1	Rr.KRYVcEIdL
AT1G68550.1	RkmKRIVrEInF
Os09g13940.1	KkeRRFSrEIlI
AT4G27950.1	RrvKRLInEIrV
AT1G25470.1	RkvKRFVhEItF
AT1G49120.1	KppKRYVsKIcV
AT4G23750.1	RrvKKFVnEVyL
AT1G22985.1	RrgKKLVkEIvI
AT2G46310.1	Rv.KRYVdEIrF
AT4G11140.1	RrvKKYVkEVvL
AT5G53290.1	RrvKRFVnEItV
AT3G61630.1	Rv.KRYVkEIrF
Consensus sequences	[KR]-x(0,2)-[KR]-[KR]-[YIFL]-[VFIS]-x-[EK]-[VLI]-x-[IVLF]

VI-L C1*

AT1G25470.1	FLt.Dsnil.LdDYsllenDIn...FsrfEnsl.P
Os01g04020.1	VLftEdml..LgDFaeed.DLdlddIgd.DfcedF
AT3G25890.1	LLteEnnnqmLdDYcgid.DLdi..Igl.Ecdg.P
AT1G68550.1	FLadDq....FdDFglld.DIqg..Fe..Dng..P
Os08g27220.1	FL-dDn..d.M.DFklssdDLs...Fl.fDig..F
Consensus sequences	[FLV]-L-x(1,2)-[DE]-x(1,5)-[MLF]-x(0,1)-D-[FY]-x(4,5)-D-[LI]-x(1,4)-[FI]-x(1,3)-[ED]-x(2,4)-[FP]

VI-L C2*

AT1G25470.1	LPdc.DFt..EM
Os01g04020.1	IPsgyDFgrgDM
AT3G25890.1	LPdy.DFs..DV
AT1G68550.1	LPdf.DFa..DV
Consensus sequences	[LI]-P-x(2,3)-D-F-x(1,3)-[ED]-[VM]

VI-L C3*

AT1G49120.1	LLDfdpliDdFkleDfPmdDlglLgD
AT2G46310.1	LPDlfrsgDyFwdsE.ItpDpl.FlD
AT5G53290.1	PLDtpfl.DnYfn.EsPp.Ems.IfD
AT4G27950.1	PLDitfl.DnYfn.EsLp.Dis.IfD
AT3G61630.1	LPDlslagEcFwdtE.IapDll.FlD
Consensus sequences	[LMP]-[LAP]-D-x(4,5)-[DE]-x-[FYI]-x(2,3)-[DE]-x(0,1)-[PLI]-x(1,2)-[ED]-x(2,3)-[LFI]-x-D

VI-L C4

AT1G68550.1	SalpDfd..FaDvedlqLa.D
AT3G25890.1	SelpDyd..FsDve...I..D
AT4G27950.1	SeikEigssFnDlddslIs.D
AT4G23750.1	SwhmEdh..FqDigdl.FgsD
AT1G25470.1	SttkEvfttF.DfsdvkIp.D
AT2G46310.1	SwdvDef..FqDhl...L..D
AT4G11140.1	SwhvEdh..FqDigdl.FgsD
AT5G53290.1	SsikDmgstFsDfddslIs.D
AT3G61630.1	SspwDvdhfF.DhhhhsF..D
Consensus sequences	S-x(3)-[DE]-x(2,4)-F-x(0,1)-D-x(2,5)-[LFI]-x(0,2)-D

Group VI

VI N1

Os06g06540.1	VRVLcrDhDaTDS.SgdDD
Os01g46870.1	VRIFwdDpDlTDS.SgeDE
Os07g12510.1	VRIIfrDeDaTDSsSseDE
Os03g60120.1	VRILvrDaDaTDSsSseDE
Os01g12440.1	VRVFcdDfDaTDSsSdeDE
Os05g25260.1	VRVYfvDaDaTDTdSsgDE
Consensus sequences	VR-[VI]-[LFYI]-x(2)-D-x-[DE]-x-TD-[ST]-x(0,1)-S-x(2)-D-[DE]

VI C1*

Os06g06540.1	DgFfsDVggd.Dl..FaAeP.F
Os01g46870.1	DyFq.DLr...Dl..FpLnP.L
Os05g25260.1	E.F..DWlpwwEgedF.VtAgL
Os01g12440.1	DdFfqDIt...Dl..FqIpV.V
Consensus sequences	[DE]-x(0,1)-F-x(0,2)-D-[WVLI]-x(1,4)-[DE]-x(1,3)-F-x(0,1)-[ALIV]-x-[PVA]-x(0,1)-[FLV]

Subgroup VIIa

VIIa N1

Os02g54160.1	MCGGAIIhhLkghP
AT3G16770.1	MCGGAIIsdYa..P
Os03g08470.1	MCGGAILaeFipaP
Os05g29810.1	MCGGAIIadFvp.P
Os06g09390.1	MCGGAILsdLippP
AT2G47520.1	MCGGAIIsdFi..W
Os09g26420.1	MCGGAIIsgFip.P
Os07g42510.1	MCGGSILgdLhl.P
AT3G14230.1	MCGGAIIsdFippP
AT1G53910.1	MCGGAIIsdFippP
Consensus sequences	MCGG-[AS]-I-[IL]-x(2)-[FLY]-x(1,3)-[PW]

VIIa N2*

Os02g54160.1	RRATeglLW
AT3G16770.1	RKLTaeeLW
Os03g08470.1	KRVTashLW
Os06g09390.1	RRVTagdLW
AT3G14230.1	LRVTnefIW
Os09g26420.1	RRVTadvLW
Os07g42510.1	RTVNagdLW
AT1G53910.1	RRVTsefIW
Consensus sequences	[KRL]-[RKT]-[VAL]-[TN]-x(3)-[LI]-W

VIIa N3

Os02g54160.1	DEDFEADFeeF
Os03g08470.1	VDDFEAAFeqF
Os06g09390.1	EDDFEADFeeF
Os09g26420.1	EEDFEADFreF
Os07g42510.1	DDDFEADFeeF
AT3G14230.1	DDDFEADFqgF
AT1G53910.1	DAEFEADFqgF
Consensus sequences	[DEV]-[EDA]-[DE]-FEA-[DA]-F-x(2)-F

VIIa C1

Os02g54160.1	EPyMrFL
AT1G72360.1	ENqTeYF
Os03g08470.1	DPfMlFQ
Os06g09390.1	EPyMkFL
AT2G47520.1	EDyMrFY
Os09g26420.1	DPeMkYL
AT3G14230.1	DDlMaYL
Consensus sequences	[DE]-[PND]-x-[MT]-x-[FY]-[FYLQ]

VIIa C2*

Os02g54160.1	LWSFDDM
Os03g08470.1	LWSFDDF
Os06g09390.1	LWSFDDM
AT3G14230.1	LWSLDEI
Os09g26420.1	IWSLDEL
Os07g42510.1	LWSFDDM
AT1G53910.1	LWSIDEI
Consensus sequences	[LI]-WS-[FLI]-D-[ED]-[FMLI] Extended LWSY motif

Subgroup VIIb

VIIb N1

AT5G61890.1	KRpfggDeseer...EEdEnlFpvFsarsqhD
Os09g11480.1	KRggggD........DDwEaaFreFiagdddD
Os09g11460.1	RRklavD........EDwEaaFreFlsrdhdD
AT5G07310.1	KRpfrgDesdekkeaDDdEniFpfFsarsqyD
Consensus sequences	KR-x(4)-D-x(0,8)-[DE]-[ED]-x-E-x(2)-F-x(2)-F-x(6)-D

VIIb C1*

Pp1s149_253V6.1	PIqtLFqqss.....SrveKsltlsvPalkvsstq..WSpG
Pp1s90_184V6.1	PVqtLFqdamgiaskSvk.KplthsvPvlevspsq..WSmD
Pp1s124_33V6.1	PIqtLFqdtmgktsySak.KpsilavPpvrtsaip..WSlG
Pp1s127_75V6.1	PIhaLFeeaghvve.SkrqRvmkkptPitpter....LSvG
Pp1s105_212V6.1	PIqtLFqesli....Slk.KplihsvPavdvsvsqveWSlG
Pp1s487_24V6.1	PIqvPFeeaghvad.SkrqRtlkkptPitpteg....LSvG
Consensus sequences	P-[IV]-x(2)-[LP]-F-x(4,9)-S-x(2,3)-[KR]-x(6)-P-x(6,10)-[LW]-S-x-[GD]

VIIb C2*

Pp1s149_253V6.1	VFLNSDnPetkP......R
Pp1s90_184V6.1	VILNSEsPeivAallq..K
Pp1s124_33V6.1	VIVNSEsPeivAtpqqrtK
Pp1s127_75V6.1	VILNSEaAcatPptphdaK
Pp1s105_212V6.1	VILNTEsPetkPts....R
Pp1s487_24V6.1	VILNSEaAcatPptphdaK
Consensus sequences	V-[FI]-[LV]-N-[TS]-[ED]-x-[AP]-x(3)-[AP]-x(0,6)-[KR]

VIIb C3*

Pp1s149_253V6.1	pFLIDLNfPPcl.D
Pp1s90_184V6.1	pFLLDLNfPPssdD
Pp1s124_33V6.1	pFLLDLNsPPcldE
Pp1s127_75V6.1	pFLLDLNqPPck.D
Pp1s105_212V6.1	sILLDLNfPPchdE
Pp1s487_24V6.1	pFLLDLNqPPcslE
Consensus sequences	[FI]-L-[LI]-D-L-N-x-P-P-x(2,3)-[ED] EAR-like Motif

Subgroup VIIc

VIIc N1*

Os03g08500.1	MCGGAILAeLiPsa.PaaRrvTAghVWP
Os03g08460.1	MCGGAILAdFtParvP..RrlTAaeLLP
Os03g08490.1	MCGGAILAnIiPatpP..RpaTAahVWP
Consensus sequences	MCGGAILA-x-[LIF]-x-P-x(2,3)-P-x(0,2)-R-x(2)-TA-x(2)-[VL]-[WL]-P

VIIc C1*

Pp1s61_230V6.1	EDdPylvsd..MArgMLiqPPsysddd.DS
Pp1s83_10V6.1	DDnPynqvde.MArgLMirPPnndsddsDD
Pp1s1_34V6.1	EDqFdverdl.YNalLIppPYyndtrd.DS
Pp1s199_50V6.1	DDdPynqvhe.MArgMMmqPPksdsddsDE
Pp1s296_32V6.1	DEiVsrssasdWFt.PFedPWcsnhal.ED
Pp1s26_306V6.1	DDdPrnqmnr.IAqaMLidPPnhdsddtDE
Pp1s45_159V6.1	DEdPlqatwe.VPdeMMv.PLtprevlyDS
Consensus sequences	[ED]-[ED]-x-[VPF]-x(5,7)-[WVIMY]-[FPAN]-x(1,2)-[PML]-[FMLI]-x(1,2)-P-[WLPY]-x(4,7)-[ED]-[EDS]

VIIc C2

Pp1s83_10V6.1	EDPPLcW
Pp1s26_306V6.1	DDPPL.W
Pp1s61_230V6.1	NGPSL.W
Pp1s1_34V6.1	GGFSL.W
Pp1s199_50V6.1	GDPPL.W
Pp1s296_32V6.1	TDFPY.L
Pp1s45_159V6.1	DDLPP.W
Consensus sequences	[TEDGN]-[DG]-[FLP]-[PS]-[YPL]-x(0,1)-[LW]

VIIc C3*

Os03g08500.1	ALlFaDQYnhfnggayeSL
Os06g47590.1	PLpFdLNMpppeeeld.MA
AT4G34410.1	MMmMdFGNgdssdsgn.TI
Os04g52090.1	GVgLdLNLpppie....EA
Os04g34970.1	PVrPlFHGgngweqrgdSA
Os03g08460.1	PLsMaLQLqyaa.....MI
Os03g08490.1	PFlFgDQFgdlnggafaSL
Os02g06330.1	PLpFdLNLpassp....SA
Consensus sequences	[LFVM]-x-[FPML]-x-[FLD]-[QHGN]-[YFGNML]-x(4,9)-[STEM]-[LAI]

Subgroup VIIIa

VIIIa N1

Pp1s7_481V6.1	LKREYtlSss.D
Pp1s293_60V6.1	LKRDYssSsddE
Pp1s293_53V6.1	VKREY..SphgE
Pp1s126_30V6.1	VNSDYccSss.D
Pp1s21_239V6.1	LKREYssSssdD
Pp1s7_472V6.1	LKREFvySsseE
Pp1s21_247V6.1	LKREFvsSde.E
Consensus sequences	[VL]-[KN]-[RS]-[DE]-[FY]-x(0,2)-S-x(2,3)-[ED]

VIIIa N2*

Pp1s7_481V6.1	KRvrVICTDPDATDSSsDEeDn.F
Pp1s293_60V6.1	KRvrVICTDPDATDSSgDEeDs.F
Pp1s293_53V6.1	KRvrVICTDPDATDSSsDEeDt.I
Pp1s126_30V6.1	KR..VICTDPDATDSSsDEkDn.F
Pp1s21_239V6.1	KRvrVICTDPDATDSSsDEeDn.F
Pp1s7_472V6.1	KRvrVICTDPDATDSSsDEeEs.F
Pp1s21_247V6.1	KRvrVICTDPDATDSSsDE.EgsF
Consensus sequences	KR-x(0,2)-VICTDPDATDSS-x-DE-x(0,1)-[ED]-x(1,2)-[FI]

VIIIa N3*

Pp1s7_481V6.1	LRnsqqR.LvqEIytstes..Favness......SDsELd...EpEmpSYH
Pp1s293_60V6.1	ARnphkR.FvqEIqmesqs..Itedefs......SDnEFn...EtEtqSSH
Pp1s293_53V6.1	VRnsqrR.LvrEIhlsset..Faaeess......SDsDLd...EpEvpSYH
Pp1s126_30V6.1	VRnphrR.Lq.EIhist....Mvdesa.......SDsEVd...ElEvpSYH
Pp1s21_239V6.1	LRnsqrR.LvqEIhmsne...Fgaadels.....SDsELd...EpEvpSYH
Pp1s7_472V6.1	MRssq.RvLvqEIdlsragseFmfmaesssa...SDsELdeleEsEvpSYH
Pp1s21_247V6.1	MRssq.RvLvqEIdmfragpeLmamaksssssssSDsEFdeteEcEvpSYH
Consensus sequences	R-x(3,4)-RK-x(0,1)-[LF]-x(1,2)-EI-x(4,8)-[LFMI]-x(5,12)-SD-x-[DE]-[FLV]-x(1,4)-E-x-E-x(2)-S-[YS]-H

VIIIa N4*

Pp1s7_481V6.1	SpnFYdep.WeS.K.KnapRVlmKkV
Pp1s293_60V6.1	SsnFYdks.WqS.RlKap.KVseKkV
Pp1s293_53V6.1	SsnFYeks.WqS.K.RiapKMseKkV
Pp1s126_30V6.1	SsrFYkks.WqSqK.Kal.KMpeKkM
Pp1s21_239V6.1	SssFYdkp.WqSkK.Kap.KAteK.V
Pp1s7_472V6.1	SasFYynpsWkSkK.Ks..KVtqKtP
Pp1s21_247V6.1	S..FYekplWkTmK.Ka..KVpgKtM
Consensus sequences	S-x(0,2)-FY-x(3,4)-W-x-[TS]-x(0,1)-[KR]-x(0,1)-[KR]-x(1,3)-[KR]-[VMA]-x(2)-K

VIIIa C1

AT1G53170.1	LdLDLNlAPP
Os05g41780.1	FdLDLNcPPP
AT3G15210.1	LdLDLNlPPP
Os05g41760.1	LpFDLNePPP
AT1G50640.1	FqFDLNfPPL
AT3G20310.1	FqFDLNfPPL
AT1G28370.1	LdLDLNfPPP
AT5G44210.1	LnLDLNlAPP
Os01g58420.1	FdLDLNrPPP
Consensus sequences	[LF]-x-[LF]-D-L-N-x-[AP]-P-[LP] EAR Motif

VIIIa C2*

Pp1s7_481V6.1	MqScCdLgqvFLsDdFLFDFP
Pp1s293_60V6.1	VqSaCdLgevFLsDnFLFDFP
Pp1s293_53V6.1	VqSaCdLgdvFLsD.FLFDFP
Pp1s126_30V6.1	VqSgSgLgevFLsDdFMMDWA
Pp1s21_239V6.1	VeScCdLgevFLsDdFLFDFP
Pp1s7_472V6.1	VqSgCdLdisLLpEdIFFDLP
Pp1s21_247V6.1	VqSgCeLdevFLsDdLFFDFP
Consensus sequences	[VM]-x-S-x-[CS]-x-L-x(3)-[FL]-L-x-[DE]-x(0,1)-[FLI]-[FLM]-[FM]-D-[FLW]-[AP]

VIIIa C3*

Pp1s7_481V6.1	VDFsaGFdcFg.GD.IgdLaFgsdqnEsLdWFnya.DIL
Pp1s293_60V6.1	LDIstGFs.FleGD.LgnLaFdcdenEsMnWLnis.DVL
Pp1s293_53V6.1	LEIaaGFd.FlgGD.LgdLaFecdysEgLdWLnst.DIL
Pp1s126_30V6.1	VEFgaGLe.Fl.GDdVdyLeFdcdgrEsFdWLngs.DIL
Pp1s21_239V6.1	LDFaaGLe.FleGD.IgdLaFgsdqnEsLeWLnaa.DIL
Pp1s7_472V6.1	LDFasGFd.F..GDvIgdFeFgeaetDsLaWFnsa.DML
Pp1s21_247V6.1	LDFavGFd.F..GDdIgdFeFgaaesDsLdWFsnaaDMP
Consensus sequences	[LV]-[DE]-[FI]-x(2)-G-[FL]-x(1,2)-F-x(0,2)-GD-x(0,1)-[IVL]-x(2)-[FL]-x-F-x(5)-[DE]-x-[LFM]-x-W-[FL]-x(3,4)-D-[MIV]-[LP]

Subgroup VIIIb

VIIIb C1*

AT5G13910.1	YVHSPMFSrMP
Os04g32790.1	YVHSPLFSpMP
Os02g32040.1	YVHSPMFSpMP
Consensus sequences	Y-V-H-S-P-[ML]-F-S-x-M-P

VIIIb C2

AT5G13910.1	DSFpQgFnyfGS
Os04g32790.1	DGF.Q.LggsSS
Os02g32040.1	DGF.Q.LggsSS
AT1G28160.1	EGF.NsLp..ST
Consensus sequences	[ED]-[GS]-F-x(0,1)-[NQ]-x(0,1)-[FL]-x(1,3)-[GS]-[TS]

Subgroup IXa

IXa N1

AT5G47220.1	DyaLLEsItrHLL
AT4G17500.1	DyaFLEsIrrHLL
AT5G51190.1	DqsALDlItqHLL
AT5G61600.1	EalAIDfIsqHLL
Consensus sequences	[ED]-x(2)-[FLA]-[IL]-[ED]-x-I-x(2)-H-L-L

IXa C1

Pp1s181_69V6.1	FLEe.DMmlnnlsGF
Os07g47790.1	FMDy.DAvma...GF
Pp1s15_234V6.1	FVDsdDMfvvglsSL
Pp1s41_212V6.1	YVDsdDMfaigltSL
Pp1s14_458V6.1	YVDadDVftl...GL
Os10g25170.1	VFDpyDVhggla.SY
Pp1s276_50V6.1	FVDseDMfti...GF
Consensus sequences	[VFY]-[FMLV]-[DE]-x(1,2)-D-[MVA]-x(3,6)-[SG]-[YFL]

IXa C2*

Pp1s181_69V6.1	DgMCLVPPptamVaET
Pp1s15_234V6.1	NaMCIPPPdpehApEA
Pp1s41_212V6.1	DaMCIPPPdsehAtET
Pp1s14_458V6.1	DaMCIPPPdsehAtET
Pp1s276_50V6.1	DaMCIPPPapehAtET
Consensus sequences	[DN]-x-M-C-[LI]-[PV]-P-P-x(4)-[VA]-x-E-[TA]

IXa C3*

Pp1s181_69V6.1	DGDeattynWEPrLWSF
Pp1s15_234V6.1	DGEegsst.WEPhLWSY
Pp1s41_212V6.1	DGEegsnt.WEPhLWSY
Pp1s14_458V6.1	DGEegsnt.WEPhLWSY
Pp1s276_50V6.1	DGEegsti.WEPhLWSY
Pp1s132_34V6.1	SDEgtsaysWEPrLWSF
Consensus sequences	[DS]-[DG]-[ED]-x(5,6)-W-E-P-x-L-W-S-[FY] LWSY motif

Subgroup IXb

IXb N1*

Os04g46240.1	MDFpgda.EdFaLeFIrEhLLGgD
Os02g43820.1	MDFsgdidDlIsLqLLhDqLLGvE
Os04g46250.1	MDFhhgdaDdFaLeFIrEhLLGvD
Consensus sequences	MDF-x(4,5)-[DE]-x-[IF]-x-L-x-[LF]-[LI]-x-[DE]-x-LLG-x-[DE]

IXb N2*	
Pp1s58_260V6.1	MaskyprKCrEha....SPgvggR
Pp1s243_70V6.1	MvgaswnKCrRtsslrsSPrtagR
Pp1s79_169V6.1	MgskiprRCaEqa....SPrgrrR
Consensus sequences	M-x(6)-[KR]-C-x-[RE]-x(2,6)-S-P-x(4)-R
IXb C1*	
Os04g46240.1	P.SsWSWDQYwD
Os01g21120.1	PaGlWTFEDY.E
Os02g43820.1	P.SsWSWDQL.E
Os04g46250.1	P.SsWSWEQYwE
Consensus sequences	P-x(0,1)-[GS]-x-W-[TS]-[FW]-[ED]-[DQ]-[YL]-x(0,1)-[ED]
IXb C2*	
Pp1s58_260V6.1	LdDeLseIpnFvgE..LDheaMdfsshGEvYY
Pp1s243_70V6.1	LpDnLqeIs.Fc.D..FDspsLssenyGDvYF
Pp1s79_169V6.1	LdDdLasIeaLmyEfdFDqeaMdcnifGDgYY
Consensus sequences	L-x-D-x-L-x(2)-I-x(1,2)-[FL]-x(1,2)-[DE]-x(0,2)-[FL]-D-x(3)-[LM]-x(5)-G-[ED]-x-Y-[FY]
**Subgroup IXc**	
IXc N1	
AT4G18450.1	M.EmdcnpnYYrmnnsnTsdplr...SS
Os07g22730.1	M.EqhshcwYYgggmavTaaagagaaSS
AT2G31230.1	M.Eysqss.MYssps..Sws......SS
AT3G23230.1	MdDyslrp.PYassss.Ss.......SS
Pp1s265_50V6.1	M.DqndsddMFiysilkDashsaad.SS
Os07g22770.1	MaEhfshagMYigyta.DaaasssssSS
AT1G06160.1	M.Eyqtn..FLsgefspEnsssssw.SS
Os09g39850.1	MsDrdvdmhILtalma.DmadsssssSS
AT3G23240.1	M.DpfliqsPFsgfsp.Eysig....SS
Consensus sequences	M-x(0,1)-[ED]-x(4,6)-[YIMFP]-[YLF]-x(2,6)-[EDST]-x(1,8)-S-S
IXc C1	
Os07g22730.1	PVLaLKgRHSkRkR
AT2G31230.1	PVMaLKrKHSlRnR
Os05g37640.1	PVLtLKrRHRiRkR
Os07g22770.1	PVLaLKrRHSkRkR
AT1G06160.1	PVIaLKrKHSmRnR
Os09g39850.1	PVLaLKrRHSiRkR
AT3G23240.1	PVVaLKrKHSmRrR
Consensus sequences	PV-[MLI]-x-LK-x-[RK]-HS-x-R-x-R Postively charged Motif
IXc C2	
AT4G18450.1	FEFEDLGsdYLETLL
Os07g22730.1	LELEDLGadYLEELL
AT2G31230.1	VVFEDLGaeYLEQLL
Os09g39810.1	IEFEYLDdhVLQEML
AT3G23230.1	FEFEYLDdkVLEELL
Os10g30840.1	IEFEYLDdkVLDDLL
Os07g22770.1	VELEDLGaeYLEELL
AT1G06160.1	VVLEDLGaeYLEELM
Os09g39850.1	VELEDLGadYLEELL
AT3G23240.1	VVFEDLGeqYLEELL
Consensus sequences	[FVLI]-[EV]-[FL]-E-[DY]-L-[GD]-x(2)-[YV]-L-[EDQ]-[EDQ]-[LM]-[LM] Extended EDLL Motif
IXc C3*	
Pp1s19_272V6.1	SEPaFRHMYETVERLASAISDPRPR
Pp1s345_35V6.1	SEPaYRHMYETVERLASAISDPRPR
Pp1s20_184V6.1	SEP.YKHMYESVERLASAISDPRPR
Pp1s121_50V6.1	SELaYRHMYESVERLASAISDPRPR
Consensus sequences	SE-[PL]-x(0,1)-[FY]-[KR]-HMYE-[ST]-VERLASAISDPRPR
**Subgroup IXd**	
IXd C1	
Os03g05590.1	VELecLDDkVLeDLL
Os04g18650.1	VELvyLDDkVLdELL
AT1G04370.1	FEFeyLDDsVLdELL
AT5G43410.1	FEFeyLDDsVLeELL
Os08g44960.1	IELecLDDqVLqEML
Consensus sequences	[VIF]-E-[LF]-x(2)-L-D-D-x-V-L-x-[ED]-[LM]-L EDLL Motif
**Subgroup IXe**	
IXe N1*	
Os03g07940.1	MkhmTRqEFIahLRRnSSG
Os07g03250.1	MknmTRqEYVahLRRkSSG
Os08g34360.1	MegqSReEYIgsLRRkSSG
Os01g59780.1	MqrmTReEYLatLRRkSSG
Os06g44750.1	MksmTRqEFIasLRRkSSG
Consensus sequences	M-x(3)-[ST]-R-x-E-[YF]-[LIV]-x(2)-L-R-R-x-S-S-G
IXe C1	
Os03g07940.1	LPvFalWn.....D
Os07g03250.1	MPlFaaWt.....D
Os08g34360.1	AVaYd.Iaaieh.R
Os01g59780.1	AVsYa.IsslasgR
Os06g44750.1	APhFynYlfgm..E
Consensus sequences	[ALM]-[VP]-x-[YF]-x(1,2)-[IFYW]-x(1,6)-[RDE]
**Subgroup Xa**	
Xa N1*	
Os02g34270.1	MaalFeAaEtaaIVaALtrVI
Os01g64790.1	MsgyYqAqEmstMVsALarVV
Os05g36100.1	LsgeYqAqEmstMVsALtwVV
Os02g34260.1	MaalFeAaEtaaIVaALtrVI
Consensus sequences	[ML]-x(3)-[FY]-x-A-x-E-x(3)-[MI]-V-x-AL-x(2)-V-[IV]
**Subgroup Xb**	
Xb C1	
Pp1s195_38V6.1	KAtrLIrlR
AT5G19790.1	KIrnLLhqK
Pp1s64_47V6.1	KAarLIrlR
Os07g10410.1	RIrtILthK
Os07g38750.1	RIrgILhhK
Os12g39330.1	KVtnLLllR
Consensus sequences	[RK]-x(3)-[LI]-[IL]-x(2)-[RK]
Xb C2*	
Os09g28440.1	LWDgLqDLmkLdea....Dt...Wfp.P
Os02g52670.1	IWDgLrEImmLd......Dgsf.Wsm.P
Os04g57340.1	VVDdAdDAstVrsrvaafDln..Lpp.P
Os08g36920.1	LWEgLqDLmqLd......Egg..LswfP
Consensus sequences	[VIL]-[VW]-[DE]-x-[AL]-x-[ED]-[AIL]-x(2)-[VL]-x(0,7)-[DE]-x(0,4)-[LW]-x(2,3)-P
**Subgroup Xc**	
Xc C1*	
AT2G35700.1	DGFWDSFPYEEPFLsqsY
AT4G16750.1	DGFWDSFPYEDPFFlenY
Consensus sequences	D-G-F-W-D-S-F-P-Y-E-[ED]-P-F-[LF]-x(3)-Y
Xc C2*	
Os04g32620.1	DLmrYAqLLqG
Os02g32140.1	DLiqYArLLrS
Os11g06770.1	EYseYArFLqG
Consensus sequences	[ED]-[YL]-x(2)-Y-A-x-[FL]-L-x-[GS]

Note that the numbers in front represent groups/subgroups, followed by N(prior to the AP2 domain) or C(after the AP2 domain). The asterisks indicate the [illegible]
