# Supplementary figures and images for: Computational Disorder Analysis in Ethylene Response Factors Uncovers Binding Motifs Critical to Their Diverse Functions
Source: Int J Mol Sci. 2019 Dec 20;21(1):74. doi: 10.3390/ijms21010074 (PMC6981732; doi:10.3390/ijms21010074)

Group I

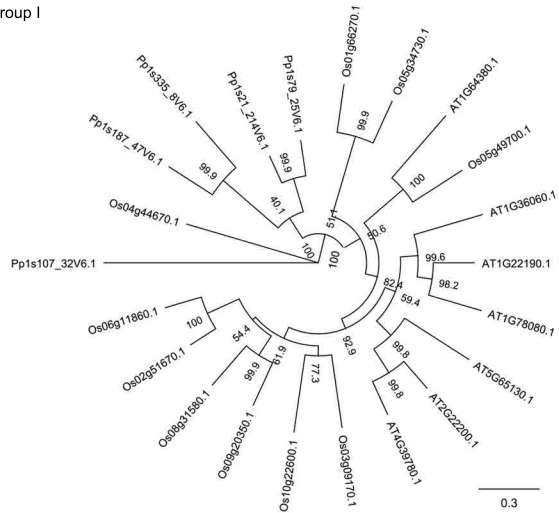

Group IIc

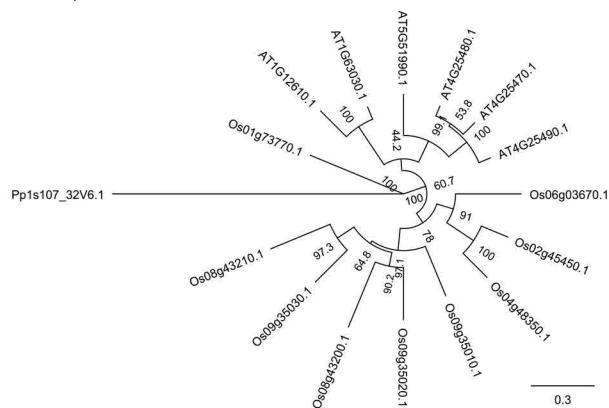

Group VIIa

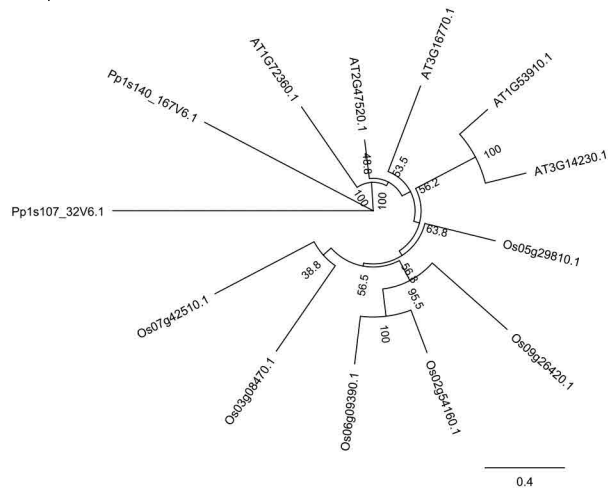

Group IIb

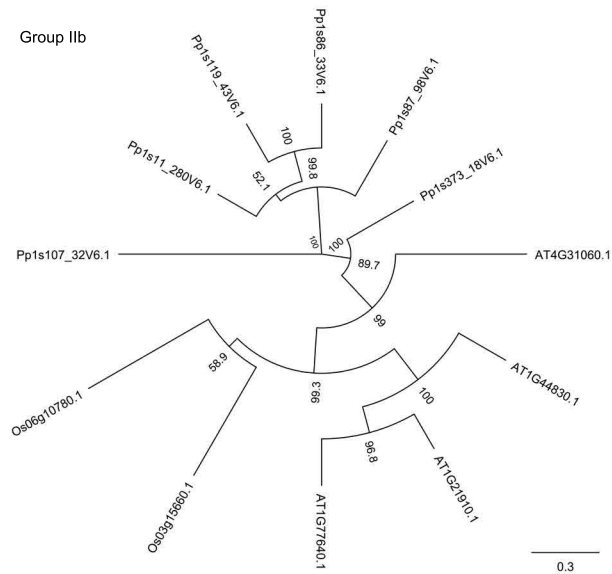

Supplement: Supplementary file 1 [file ijms-21-00074-s001.zip › ijms-650542-supplementary-XML/Figure S1.pdf]
